# SUPPRESSOR OF PHYTOCHROME B-4 #3 reduces the expression of PIF-activated genes and increases expression of growth repressors to regulate hypocotyl elongation in short days

**DOI:** 10.1186/s12870-022-03737-z

**Published:** 2022-08-15

**Authors:** Caitlin N. Jacques, David S. Favero, Ayako Kawamura, Takamasa Suzuki, Keiko Sugimoto, Michael M. Neff

**Affiliations:** 1 RIKEN Center for Sustainable Resource Science, Yokohama, Kanagawa 230-0045 Japan; 2 Department of Crops and Soil Sciences, Washington State University, Pullman, WA 99164 USA; 3 Molecular Plant Sciences Graduate Program, Washington State University, Pullman, WA 99164 USA; 4 Department of Biological Chemistry, College of Biosciences and Biotechnology, Chubu University, Kasugai, Aichi 487-8501 Japan; 5 Department of Biological Sciences, The University of Tokyo, Tokyo, 119-0033 Japan

**Keywords:** SOB3, AHL, Hypocotyl, PIF, SD, Arabidopsis

## Abstract

SUPPRESSOR OF PHYTOCHROME B-4 #3 (SOB3) is a member of the AT-HOOK MOTIF CONTAINING NUCLEAR LOCALIZED (AHL) family of transcription factors that are involved in light-mediated growth in *Arabidopsis thaliana*, affecting processes such as hypocotyl elongation. The majority of the research on the AHLs has been conducted in continuous light. However, there are unique molecular events that promote growth in short days (SD) compared to constant light conditions. Therefore, we investigated how AHLs affect hypocotyl elongation in SD. Firstly, we observed that AHLs inhibit hypocotyl growth in SD, similar to their effect in constant light. Next, we identified AHL-regulated genes in SD-grown seedlings by performing RNA-seq in two *sob3* mutants at different time points. Our transcriptomic data indicate that *PHYTOCHROME INTERACTING FACTORS* (*PIF*s) *4*, *5*, *7*, and *8* along with PIF-target genes are repressed by SOB3 and/or other AHLs. We also identified PIF target genes that are repressed and have not been previously described as AHL-regulated, including *PRE1, PIL1, HFR1*, *CDF5,* and *XTR7*. Interestingly, our RNA-seq data also suggest that AHLs activate the expression of growth repressors to control hypocotyl elongation, such as *HY5* and *IAA17*. Notably, many growth-regulating and other genes identified from the RNA-seq experiment were differentially regulated between these two *sob3* mutants at the time points tested. Surprisingly, our ChIP-seq data suggest that SOB3 mostly binds to similar genes throughout the day. Collectively, these data suggest that AHLs affect gene expression in a time point-specific manner irrespective of changes in binding to DNA throughout SD.

**Supplementary Information:**

The online version contains supplementary material available at 10.1186/s12870-022-03737-z.

## Introduction

Plants have a complex set of signaling pathways that enables them to interpret and respond to their surroundings. One mechanism by which plants alter their growth and development in response to environmental cues is through changes in gene transcription. An important group of transcriptional regulators involved in this process are members of the AT-HOOK MOTIF NUCLEAR LOCALIZED (AHL) family [1–3]. In *Arabidopsis thaliana* (Arabidopsis), AHL29/ SUPPRESSOR OF PHYTOCHROME B4-#3 (SOB3) has an important function regulating growth and development in response to light, affecting processes such as hypocotyl growth, flowering, and senescence [1, 4–10]. *SOB3* was identified as a repressor of hypocotyl growth in an activation-tagging screen for suppressors of the long-hypocotyl phenotype exhibited by the *phytochrome B-4* (*phyB-4*) photoreceptor mutant grown in white light [1, 11–13]. Further investigation revealed that SOB3 regulates hypocotyl growth in a light-dependent manner, affecting growth specifically in dim light [1]. Therefore, *SOB3-Dominant* (*SOB3-D*) seedlings, which are derived from the aforementioned activation-tagging experiment, have enhanced expression of *SOB3* and exhibit short hypocotyls compared to wild-type Col-0 plants [1, 5]. In addition to SOB3, other AHLs act in a semi-redundant fashion with this family member to regulate hypocotyl growth in light [6].

The *AHL* gene family is characterized by two conserved elements important for its function: a PLANT AND PROKARYOTIC CONSERVED/DOMAIN OF UNKNOWN FUCTION #296 (PPC/DUF296), and one or two AT-hook motif(s) [6, 14, 15]. SOB3 interacts with itself, other AHLs, and non-AHL transcription factors via the PPC domain [6]. On the other hand, the AT-hook motif(s) bind DNA sequences rich in adenine and thymine [4, 6, 16–18]. Notably, a single missense mutation within the central AT-hook domain of *SOB3* in the *SOB3-D* background, known as the s*ob3-6* allele, produces extremely tall seedlings [1]. This phenotype is attributed to a dominant-negative effect of a non-functional SOB3-6 protein being overexpressed, i.e. the SOB3-6 protein can engage in protein–protein interaction as it normally would, but cannot bind to DNA [6]. This leads to the formation of unproductive complexes that fail to inhibit seedling growth. From this research, it became clear that the AT-hook and PPC domain are both important for the function of SOB3 and other AHLs.

AHLs regulate growth in both constant light and long days (LD; 16 h light/8 h dark) at least partially by repressing the auxin pathway. In seedlings grown in constant light, SOB3 binds directly to the promoters of the auxin biosynthesis gene *YUCCA8* (*YUC8*) and auxin signaling genes belong to the *SMALL AUXIN UP RNA 19* (*SAUR19*) subfamily and represses their transcription [5]. However, overexpression of SAUR19 only partly recovers the short-hypocotyl phenotype of *SOB3-D*, suggesting that the effect of AHLs on growth is not fully explained by its effect on the auxin pathway. In a different study using seedlings grown in LD, Lee and Seo found that AHLs repress *YUC9* transcription by promoting deposition of the H2A.Z histone variant on the promoter of this gene [8]. This likely represses auxin biosynthesis and consequently, hypocotyl growth in older seedlings. Additionally, results from another study using LD-grown plants suggest that AHLs inhibit petiole growth largely by acting on the auxin pathway [18]. In this study, it was found that AHLs limits petiole elongation by antagonizing the growth-promoting PHYTOCHROME-INTERACTING FACTORs (PIFs). Specifically, AHLs repress PIF-mediated activation of genes involved in hormone-mediated growth, such as *ACS8*, which is involved in ethylene biosynthesis, *NPY1*, which is involved in auxin transport, *YUC8*, and *SAUR24*, thus accounting for their negative effect on petiole growth.

The majority of the research on AHLs has been performed in continuous white light or LD. Not only is constant light uncharacteristic of a natural growth environment, but there are likely different mechanisms that regulate growth in short days (SD; 8 h light/16 h dark), compared to continuous light or LD [18]. Additionally, it is unknown if AHLs have different effects on gene expression throughout the day. Therefore, in order to investigate these questions, we examined how AHLs regulate hypocotyl elongation in SD, a condition in which little is known about how this family of transcription factors affects growth.

## Results

### AHLs repress hypocotyl growth in SD

**Fig. 1:**
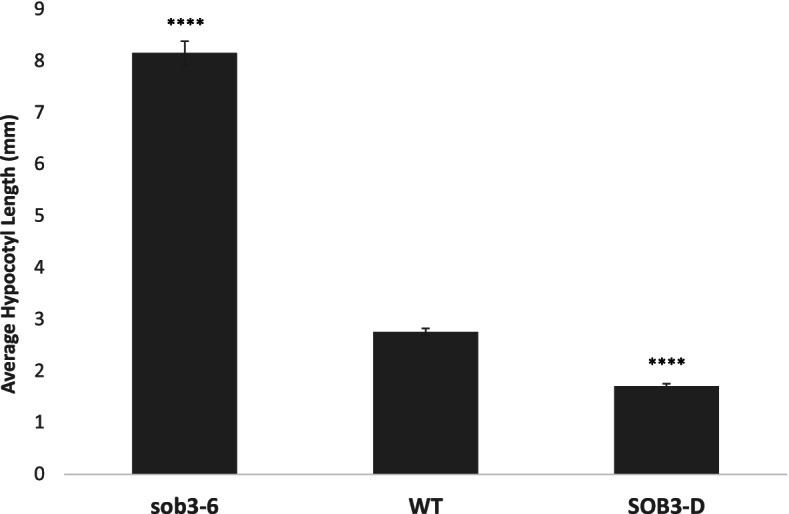
AHLs repress hypocotyl growth in SD. Hypocotyl measurements of *sob3-6*, WT Col-0, and *SOB3-*D five-day-old seedlings grown in SD. Error bars represent standard error of the mean. In a Welch’s t test (unpaired two-tailed t test with unequal variance) compared with the wild type: *P* ≤ 0.0001 = ****

We investigated how AHLs regulate hypocotyl elongation in short days (SD) (8 h light/16 h dark), a condition in which little is known about how this family of transcription factors affect growth. Phenotypic analysis of mutants for *SOB3* revealed that *SOB3-D* exhibits reduced hypocotyl growth in SD, while *sob3-6* seedlings are extremely tall (Fig. 1). These phenotypes indicate that SOB3 and/or other AHLs repress hypocotyl growth in SD, similar to what has been reported for seedlings grown in constant light or long days [1, 4–6, 8, 18].

### AHLs regulate the expression of many genes in a time point-specific manner in SD

**Fig. 2:**
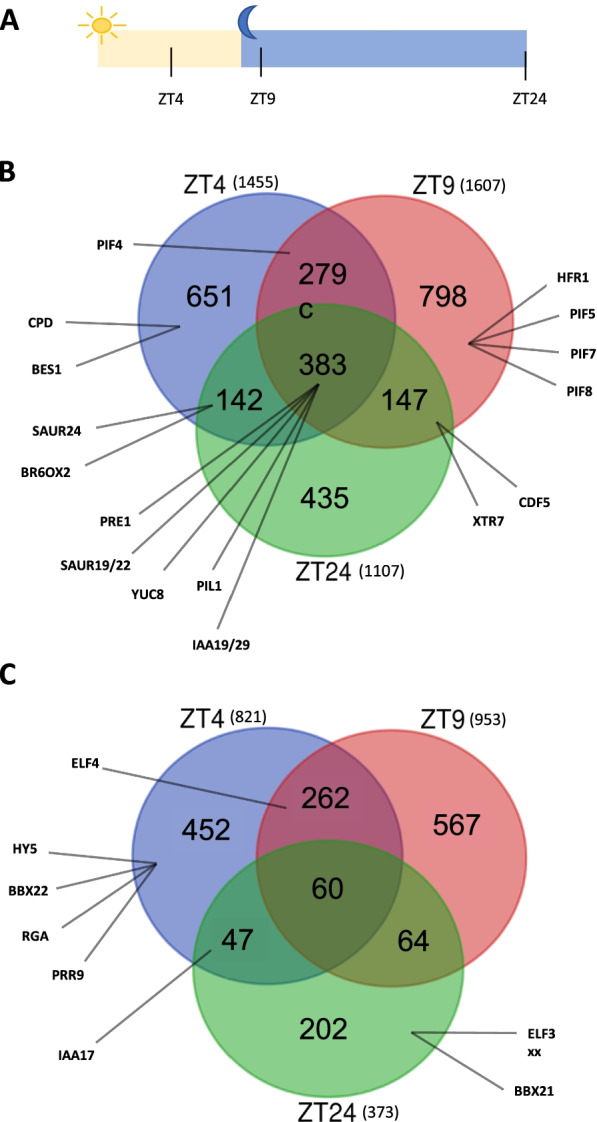
AHLs repress the expression of growth-promoting genes while also activating growth repressors. RNA-seq analysis of *SOB3-D* and *sob3-6* seedlings in SD. **A** SD photoperiod diagram depicting the time points used to harvest seedings for RNA-seq. The diagram starts at the beginning of day five and ends at the beginning of day six. **B**, **C** AHL-repressed (**B**) or -induced (**C**) nuclear-encoded genes identified from the RNA-seq data based on having higher or lower expression in *sob3-6* than *SOB3-D*, respectively. In (**B**), AHL-repressed *PIF*s, PIF-target genes, and BR-associated genes are named. In (**C**), AHL-induced growth repressors are named

Next, in order to gain insight into the molecular mechanisms by which AHLs affect hypocotyl growth in SD, we identified nuclear-encoded genes that are differentially expressed between *SOB3-D* and *sob3-6*. Specifically, we performed RNA-seq using seedlings harvested at three different time points: at ZT4 (four hours after dawn) and ZT9 (one hour after dusk) in five-day-old seedlings, and at dawn in six-day-old seedlings (ZT24) (Fig. 2A; Table S1). The three time points of ZT4, ZT9, and ZT24 were specifically chosen based on what is known about the regulation of hypocotyl growth as well as PIF activity in SD. In SD, hypocotyl growth mainly occurs at the end of the night, when high expression of *PIF4* and *PIF5* coincides with minimal post-translational repression of the *PIF*s by phytochromes and other inhibitory factors [19–21]. Therefore, we chose to test the ZT24 time point to investigate if AHLs might be preventing excessive growth at this time point by binding to and regulating the expression of *PIF*s and their target genes. On the other hand, we chose the other two time points because they represent times during the day (ZT4) or evening (ZT9) when PIF activity and growth are minimal. We reasoned that AHLs might be contributing to repression of growth at one or both of these time points, possibly by inhibiting *PIF* transcription and/or the expression of PIF-activated genes.

EdgeR analysis of our RNA-seq data for *SOB3-D* and *sob3-6* revealed 2,276 differentially expressed genes at ZT4, 2,560 at ZT9, and 1,480 at ZT24 (Fig. 2B, C), indicating that SOB3 and/or other AHLs might have a greater impact on gene expression during the middle of the daytime and early during the evening than at dawn. Of these differentially expressed genes, substantially more, 2,835, were found to be repressed by AHLs (Fig. 2B) with only 1,654 induced by AHLs (Fig. 2C). This suggests that AHLs might act more often as transcriptional repressors than transcriptional activators in SD. Furthermore, the majority of genes are mis-regulated exclusively at only one time point. Among the AHL-repressed genes, 651 are uniquely repressed at ZT4, 798 at ZT9, and 435 at ZT24, which represents 23%, 28%, and 15% of all repressed genes, respectively (Fig. 2B). Consistent with the overall smaller number of AHL-induced compared to AHL-repressed genes, there are fewer uniquely induced genes at all time points: 452 at ZT4, 567 at ZT9, and 202 at ZT24 (Fig. 2C). The uniquely induced genes at ZT4 and ZT9 make up 28% and 26% of all induced genes respectively, whereas ZT24 represents only 13%. Interestingly, there are relatively few genes mis-regulated at all three time points, particularly for the AHL-induced genes. Among induced genes, only 4% are mis-regulated at all three time points, whereas 13% are mis-regulated at all three time points for AHL-repressed genes (Fig. 2B, C). Collectively, these data suggest that the expression of most AHL-regulated genes is only affected by these transcription factors at certain times during the day.

### AHLs regulate the expression of *PIF*s and their target genes to control growth of the hypocotyl in SD

At least one way by which AHLs inhibit growth is by reducing the expression of PIF-activated genes [18]. Therefore, we investigated how the expression of several PIF target genes are affected in our RNA-seq data, focusing on PIF-activated genes highlighted in a recent review [21]. Indeed, our transcriptomic data indicate that PIF target genes are repressed by AHLs throughout the day in SD-grown seedlings (Fig. 2B). Some PIF-activated genes were identified as AHL-repressed at all three time points, such as *YUC8*, *IAA19*, and two *SAUR*s (Fig. 2B), which are all growth promoting genes associated with the auxin pathway that have been also previously identified as genes directly repressed by the AHLs [5, 18]. Notably, we found that in addition to *IAA19*, the similarly functioning and PIF-target gene *IAA29* [22–24] is also repressed by the AHLs at all three time points (Fig. 2B). The PIF-activated genes *PIL1* and *PRE1* [25, 26] are also found among the AHL-repressed genes at all three time points (Fig. 2B). Notably, PRE1 promotes hypocotyl growth, likely at least in part by enhancing PIF protein activity [26–31]. Other PIF targets were only identified as AHL-repressed at one or two time points. For example, the growth-promoting gene *CDF5* [32] is found among the AHL-repressed genes at ZT9 and ZT24, but not at ZT4 (Fig. 2B). Additionally, some genes that are differentially regulated between *SOB3-D* and *sob3-6* at all three time points exhibit different fold changes in expression at the different time points, such as *SAUR22* (greatest fold change at ZT4), *SAUR19* (greatest fold change at ZT9), and IAA19 (greatest fold change at ZT24), (Table S2). Thus, AHLs appear to have quantitatively different effects on the expression of some PIF-regulated genes throughout the day.

In addition to finding that many PIF targets are repressed by AHLs, our data also indicate that AHLs repress the expression of *PIF*s themselves in SD. Specifically, *PIF4* is among the AHL-repressed genes at ZT4 and ZT9, while *PIF5*, *7*, and *8* are repressed exclusively at ZT9 (Fig. 2B). These data suggest that AHLs regulate the expression of *PIF*s during the daytime and early in the evening, but not late in the night. Remarkably, this is in contrast to the effect of AHLs on the expression of PIF target genes described in the previous paragraph, which appears instead to persist throughout the entire day. Therefore, AHLs likely inhibit hypocotyl growth in SD at least in part by reducing the expression of PIF-activated genes, which is likely partially a downstream consequence of AHLs repressing expression of the *PIF*s themselves.

### AHLs repress brassinosteroid biosynthesis genes and activate the expression of growth repressors to regulate hypocotyl growth in SD

Our RNA-seq data also indicate that AHLs likely repress brassinosteroid (BR) biosynthesis downstream of the PIFs as well as BR signaling. *CPD* and *BR6OX2*, both PIF4-activated genes that encode enzymes involved in brassinosteroid biosynthesis [33, 34], are found among the ZT4 AHL-repressed genes, while *BR6OX2* also falls into this group at ZT24 (Fig. 2B). We further found that like *CPD*, *BES1*, which encodes a key transcription factor involved in BR-mediated hypocotyl growth [35, 36], is specifically repressed by AHLs at ZT4 (Fig. 2B). Taken together, these data suggest that in SD, the repressive effect of AHLs on hypocotyl growth is likely caused in part by them inhibiting the BR pathway.

Besides repressing the expression of genes that promote hypocotyl growth in SD, our RNA-seq data also indicate that AHLs activate the expression of growth repressors. Specifically, eight growth repressors highlighted in the Favero et al*.* review [21] are among the AHL-induced genes we identified from our RNA-seq experiment (Fig. 2C). Of these eight genes, six were identified as AHL-induced only at a single time point. Four growth repressors, including *HY5* and *BBX22*, are AHL-induced specifically at ZT4. Notably, HY5 and BBX22 proteins are protected from COP1-mediated degradation only in the presence of light [37, 38], thus AHLs appear to activate *HY5* and *BBX22* specifically when their encoded proteins are able to contribute to hypocotyl growth repression, i.e. during the daytime [39–41]. *BBX22*’s close homolog, *BBX21*, on the other hand, is one of two genes we found to be AHL-induced only at ZT24; however, BBX21 is unlikely to contribute to growth repression at the end of the night, since it, like BBX22, is largely degraded in a COP1-dependent manner in darkness [41, 42]. In addition to these six growth repressors, two were identified as AHL-induced at two different time points: *ELF4* at both ZT4 and ZT9 and *IAA17* at both ZT4 and ZT24. Collectively, our RNA-seq data suggest that AHLs inhibit hypocotyl growth in SD by not only repressing the expression of PIF-activated, growth-promoting genes, but also by increasing the expression of growth repressors, particularly during the daytime.

### Changes in SOB3 binding do not explain the differential regulation of genes at ZT4, ZT9, and ZT24 in SD

**Fig. 3:**
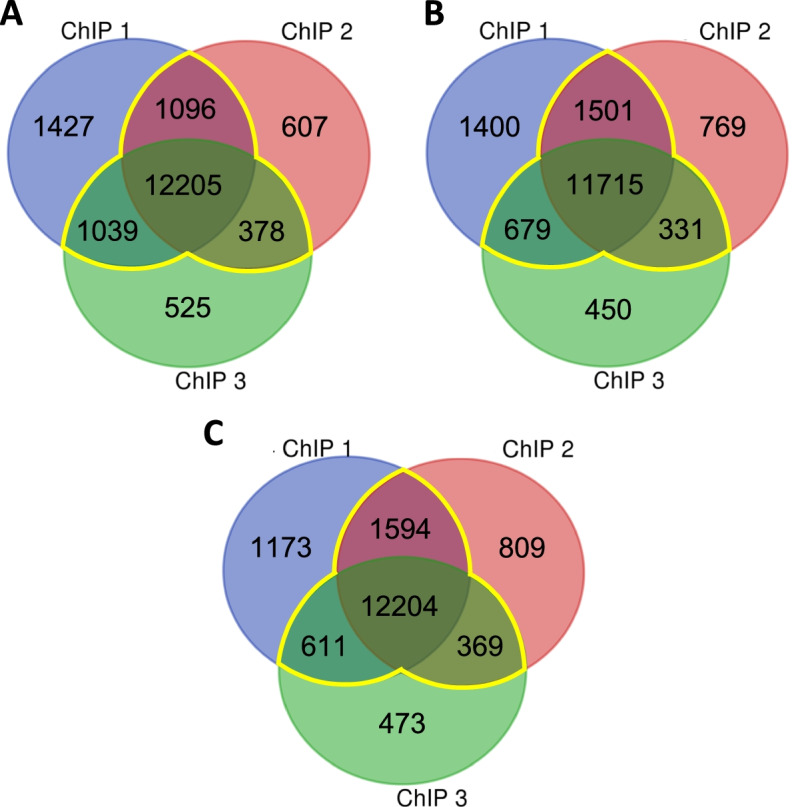
SOB3 binds a similar number of genes at ZT4, ZT9, and ZT24. Overlap between genes found to be bound by SOB3 in different replicates of the ChIP-seq experiment at A) ZT4, B) ZT9, or C) ZT24. ChIP 1 is the first replicate, ChIP 2 is the second replicate, and ChIP 3 is the third replicate. Yellow outlines indicate genes that were used for subsequent analysis, i.e. those bound in at least two replicates within a time point

**Fig. 4:**
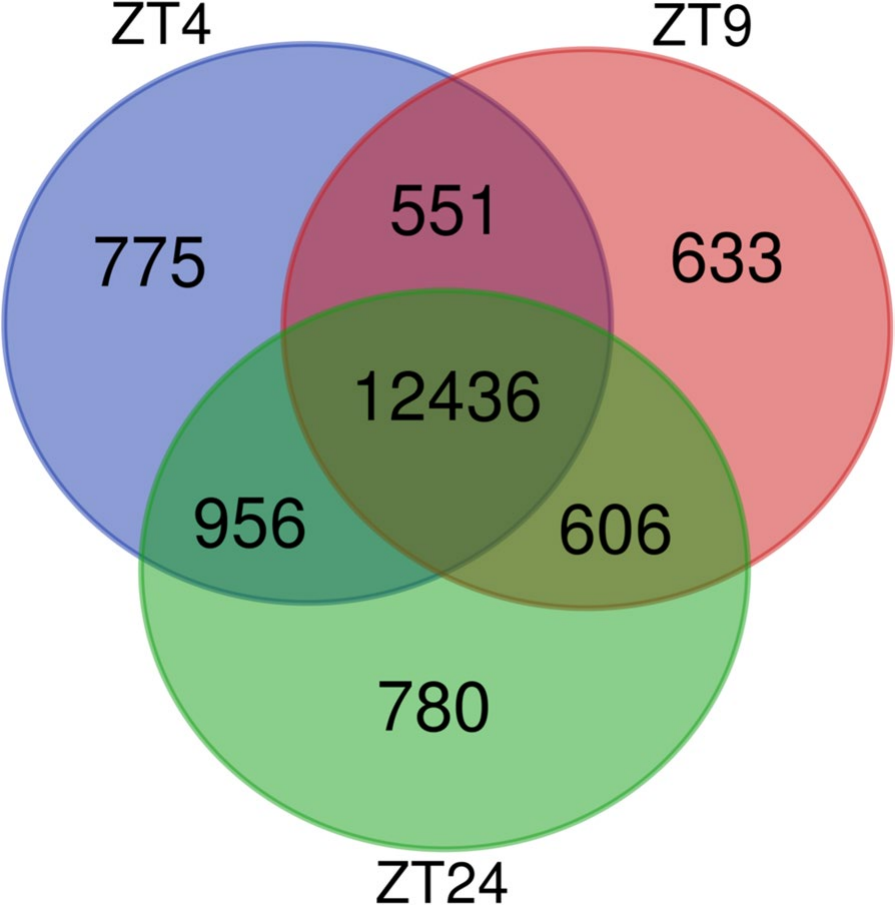
Most SOB3-bound genes are bound at ZT4, ZT9, and ZT24. Overlap between genes bound by SOB3 in at least two ChIP-seq replicates at each time point

Since our RNA-seq data show that AHLs regulate somewhat different sets of genes at different times during the day, we next investigated if SOB3 binding to DNA changes throughout the day in SD by performing ChIP-seq at the same three time points used for RNA-seq (Fig. 2A; Table S3). Genes bound by SOB3 in at least two of the three replicates for a given time point were considered SOB3-bound genes for subsequent analyses (Fig. 3, yellow outline), which resulted in 14,718 genes at ZT4, 14,226 genes at ZT9, and 14,778 genes at ZT24. When these genes were compared to each other, we found that most genes are bound by SOB3 at all three time points (Fig. 4). Interestingly, there are a smaller number of genes that are only bound at one or two time points (Fig. 4). These observations suggest that SOB3 is mostly binding to similar genes at these time three points.

**Fig. 5:**
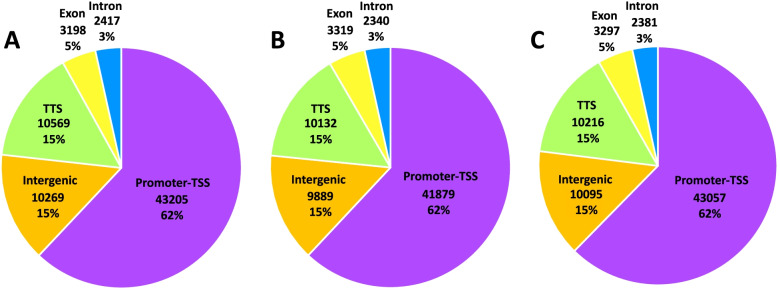
SOB3 binds the same types of genomic regions at different time points. SOB3 binding pattern distribution determined from the ChIP-seq data based on annotations obtained using HOMER software. Distribution of SOB3 binding at **A** ZT4, **B** ZT9, and **C** ZT24. Promoter-TSS is defined as -1,000 bp to + 100 bp in relation to a transcription start site. TTS is defined as -100 bp to + 1000 bp in relation to a transcription termination site

**Fig. 6:**
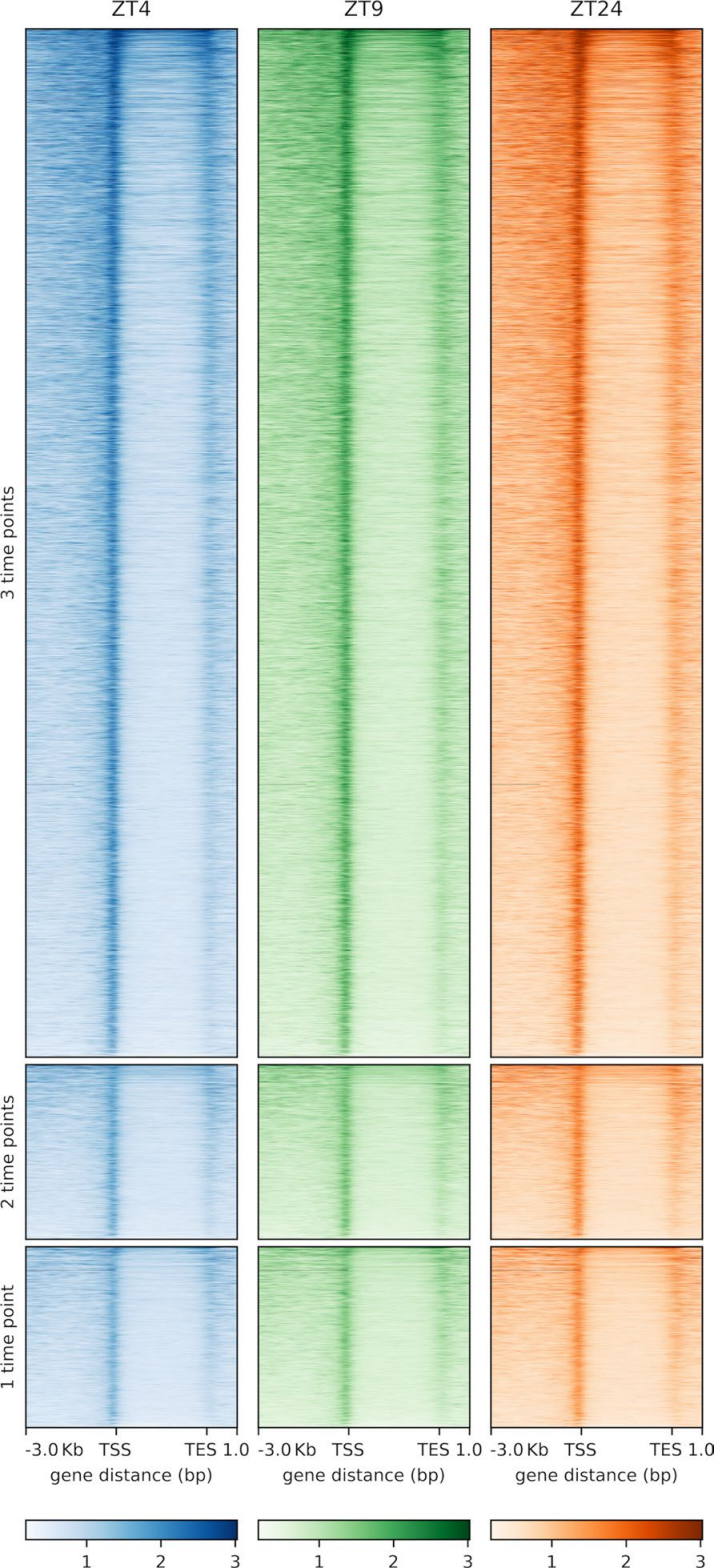
SOB3 exhibits similar binding to target genes at ZT4, ZT9, and ZT24. Distribution of SOB3 binding, as determined from the ChIP-seq data, to genes identified as SOB3-bound at one, two, or three time points

Next, we looked at the distribution of SOB3 binding sites in order to evaluate how SOB3 is functioning in the genome. We observed that the majority of binding sites are in close proximity to a transcription start site (promoter-TSS) (Fig. 5). Additionally, SOB3 has few binding events within genes bodies, with 5% of binding events occurring in exons and 3% occurring in introns. Favero et al. similarly observed that SOB3 mainly binds genomic regions located outside of gene bodies, with a high proportion of binding events occurring in promoter-TSS regions [18]. Interestingly, each time point has the same percentage of binding events in each of the five categories. The high proportion of binding events observed in promoter-TSS regions at all three time points lends further support to previous findings which indicate that SOB3 functions as a transcription factor [1, 4, 6, 14, 16, 17, 43–51], and further suggests that this role of SOB3 remains consistent throughout the day. In addition, when the binding patterns from the three time points are compared, we observe nearly identical binding of SOB3 at all three time points (Fig. 6), suggesting that this transcription factor largely binds to similar loci throughout the day. Therefore, it is unlikely that a change in binding to target genes explains the different effects of AHLs on gene expression at ZT4, ZT9 and ZT24.

### Motifs associated with TCPs and PIFs are highly enriched within the SOB3 ChIP-seq data

**Fig. 7:**
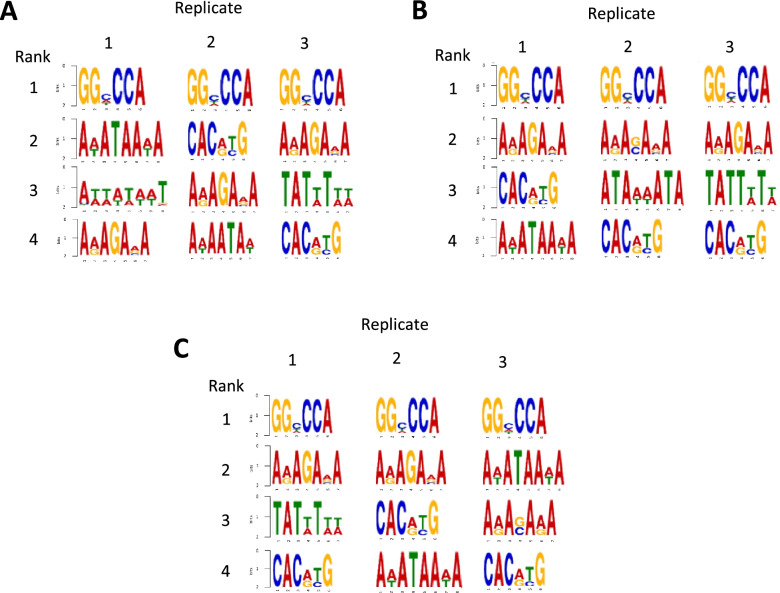
Motifs associated with TCP-binding, PIF4-binding, and AHL-binding are all enriched in regions bound by SOB3. Top 4 enriched motifs from the ChIP-seq data at each time point: **A** ZT4, **B** ZT9, and **C** ZT24. Enriched motifs were identified in the 300 bp regions surrounding SOB3 peaks using CentriMo and DREME software in MEME-Suite.

We then used MEME-ChIP to conduct motif enrichment analysis of the ChIP-seq data [52]. This analysis showed the GGHCCA motif, which resembles sequences bound by TCPs [53–56], as the most enriched motif in all replicates for all time points (Fig. 7). This motif is also much more highly enriched compared to all other motifs in every replicate and at every time point, which suggests an intimate relationship between AHLs and the TCPs in SD. The GGHCCA motif was also seen as the most enriched motif in a recent paper by Favero et al. that examined the effects of SOB3 in long days on petiole growth [18]. As noted in their paper, this motif is likely the most enriched in this data set because TCPs and AHLs have been shown to physically interact [6, 53, 54, 57]. As such, this motif is likely bound by TCPs that are in a SOB3 protein-DNA complex. Our MEME-ChIP analysis also revealed enrichment of other types of motifs at all three time points (Fig. 7). One such motif is CACRYG, which is similar to sequences bound by PIFs [25, 26, 54, 56, 58–60]. This is particularly interesting given our findings from the RNA-seq data that the expression of several PIF-activated genes are repressed by the AHLs. Therefore, it is likely that AHLs bind directly to the promoters of PIF targets and regulate their expression in SD, which also appears to be happening in the context of petiole growth [18]. Various AT-rich motifs are also highly ranked at all three time points. Some of these, including AWATAAWA, AWAATAW, TATWTWW, ATAWWATA, and TATTWTW, exclusively contain adenine and thymine residues and are similar to motifs that have been demonstrated to be bound by other AHLs, including AHL12, AHL20, and AHL25, in vitro [4, 6, 14, 16, 17, 51, 54, 56]. These motifs may be bound by SOB3 itself and/or other AHL family members found in complex with SOB3. Notably, the motifs ARAGAVA or ARASAVA are also present in all replicates from all time points and could be bound by AHLs since they are also AT-rich. However, this possibility seems unlikely, since from a structural perspective, four consecutive A/T base pairs appear to be needed to accommodate binding by an AT-hook [61].

### GO term enrichment shows AHLs directly induce genes associated with similar processes at ZT4 and ZT9, but not at ZT24

**Fig. 8:**
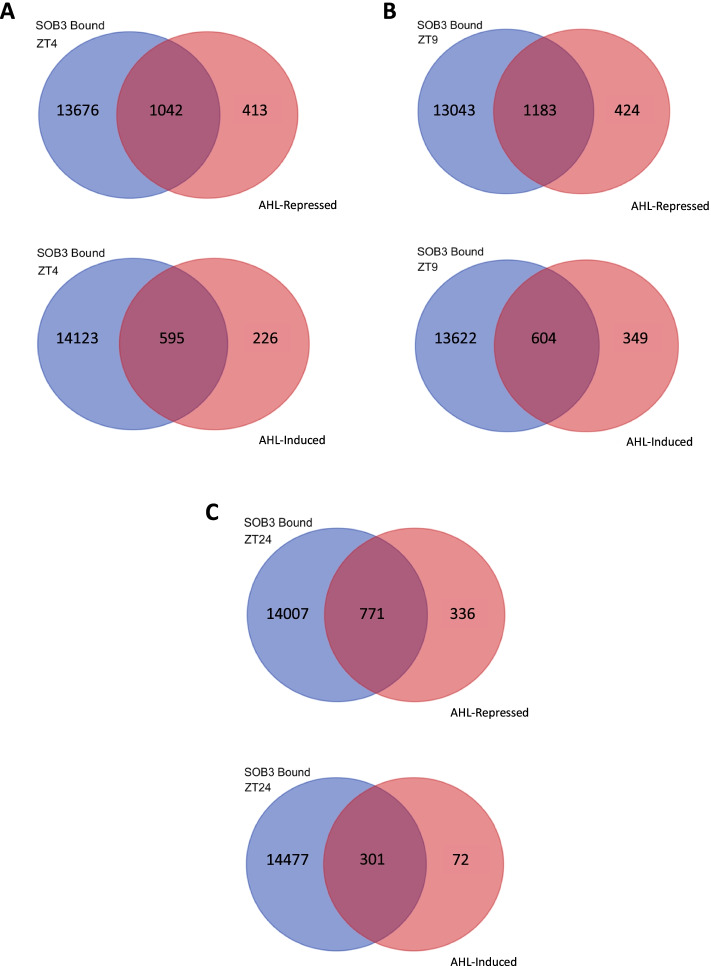
SOB3-bound genes are more frequently repressed than activated by AHLs in SD. Overlap between AHL-repressed or AHL-induced genes identified from the RNA-seq data and SOB3-bound genes identified from ChIP-seq at each of the three time points tested: **A** ZT4, **B** ZT9, and **C** ZT24

Next, to identify genes that are directly regulated by AHLs, we compared our RNA-seq and ChIP-seq results. We identified 1,042 genes as directly repressed by AHLs at ZT4, 1,183 at ZT9, and 771 at ZT24. Interestingly, we found fewer genes were directly induced by AHLs with 595 at ZT4, 604 at ZT9, and 301 at ZT24 (Fig. 8). Thus, it appears that AHLs are likely acting as both repressors and activators of transcription in SD. However, it also appears that AHLs have a bias towards acting as repressors, which is in agreement with what we had inferred earlier based on the RNA-seq data alone. Notably, this bias of AHLs towards acting as transcriptional repressors seems to be particularly high at ZT24. Comparing SOB3-bound AHL-regulated genes at each time point, we see that 64% of them are repressed at ZT4 and 66% at ZT9, while 72% of them are repressed at ZT24 (Fig. 8).

**Fig. 9:**
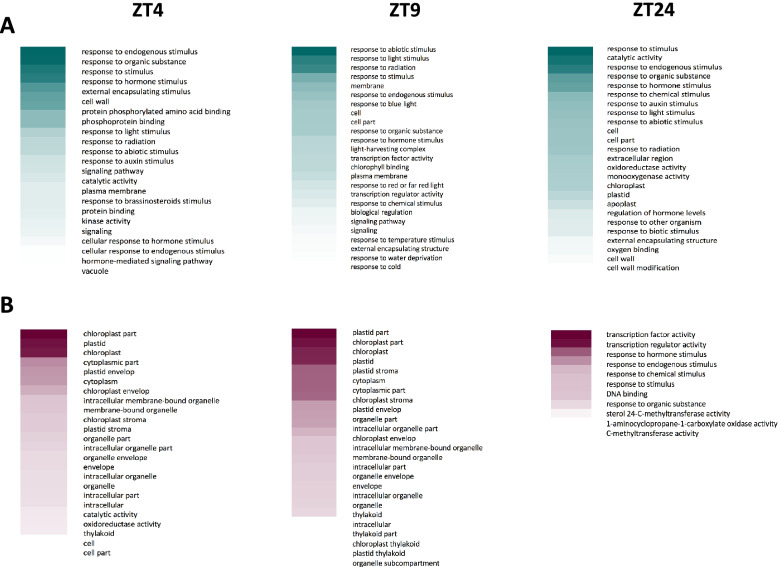
AHLs directly regulate the expression of somewhat different types of genes at ZT4, ZT9, and ZT24. Top 25 most enriched GO terms among genes identified as SOB3-bound and AHL-repressed (**A**), or SOB3-bound and AHL-induced (**B**) at ZT4, ZT9 or ZT24. The relative *P* value for each GO term is indicated by color, with darker shades representing smaller *P* values

To further investigate differences in the genes directly regulated by AHLs based on time point, we compared GO term enrichment among the different time points for the genes we identified as direct targets of AHLs. The top enriched GO terms among genes that are directly repressed by AHLs largely include those involved in responses to the external environment and by extension, the internal responses that would follow (Fig. 9). Terms such as “response to endogenous stimulus”, “response to organic substance”, “response to stimulus”, and “response to hormone stimulus” are among the top repressed GO terms for all time points. However, there are some notable differences when ZT24 is compared to ZT4 and ZT9. For example, “catalytic activity” appears as the second highest GO term among directly repressed genes at ZT24, but is much lower for ZT4 and is not present within the top 25 for ZT9. There are also other interesting time point-specific findings within the GO terms of repressed genes, such as “response to auxin stimulus,” which is highly enriched at ZT4 and ZT24, but not at ZT9. Additionally, “response to brassinosteroid stimulus”, “protein phosphorylated amino acid binding”, and “phosphoprotein binding is highly enriched at ZT4. Contrastingly, “light-harvesting complex” and “chlorophyll binding” are both highly enriched specifically at ZT9. The top GO terms among genes directly induced by AHLs for both ZT4 and ZT9 time points are “chloroplast part”, “plastid” or “plastid part”, and “chloroplast”, but the top GO terms for the ZT24 time point are entirely different and include, “transcription factor activity”, “transcription regulator activity”, and “response to hormone stimulus”. Moreover, the top functions unrelated to chloroplast function that are associated with directly induced genes at ZT24 are mostly functions that are also highly enriched among directly repressed targets at this time point. Taking these data together, it seems that there are somewhat specific processes being regulated by the AHLs at the different time points, but that ZT4 and ZT9 share more similarities when compared with ZT24.

### Changes in binding of SOB3 to target genes do not explain differential regulation of genes by AHLs at ZT4, ZT9, and ZT24

**Fig. 10:**
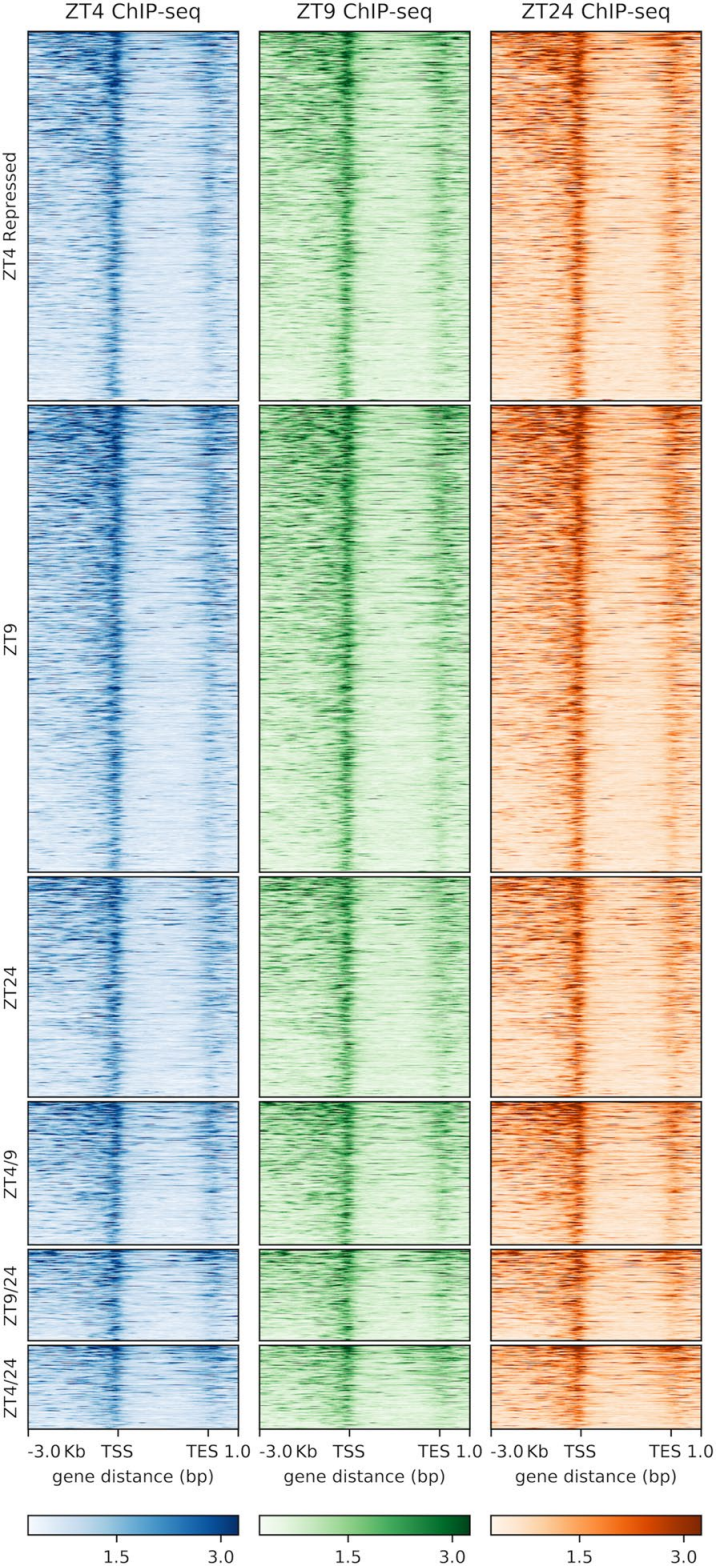
SOB3 exhibits similar binding to genes identified as directly repressed by AHLs at ZT4, ZT9, and ZT24. Distribution of SOB3 binding, as determined from the ChIP-seq data, to genes identified as both SOB3-bound and AHL-repressed at only one or two time points

**Fig. 11:**
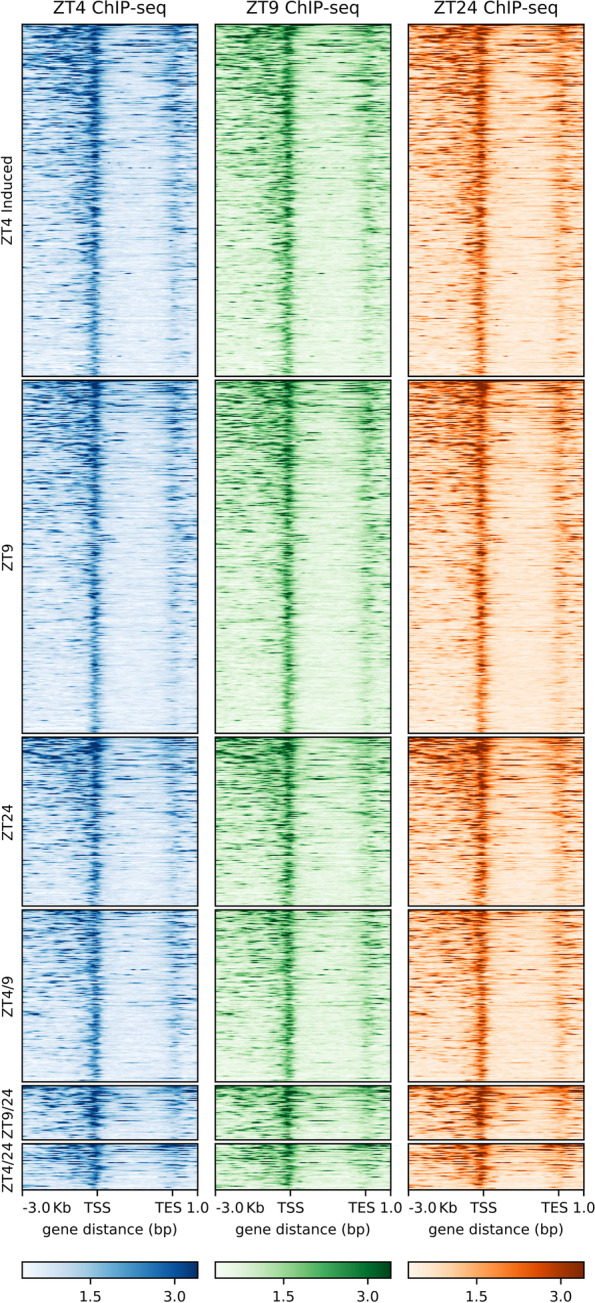
SOB3 exhibits similar binding to genes identified as directly induced by AHLs at ZT4, ZT9, and ZT24. Distribution of SOB3 binding, as determined from the ChIP-seq data, to genes identified as both SOB3-bound and AHL-induced at only one or two time points

Finally, we returned to the question of whether differences in SOB3 binding at ZT4, ZT9, and ZT24 might explain the time point-specific effects of the AHLs on gene expression. First, we examined if and at which time points the AHL-regulated genes identified from our RNA-seq data and specifically highlighted in Figs. 2B and 2C are bound by SOB3. We found that nearly all of these genes are bound by SOB3, based on our ChIP-seq data, at all three time points (Table S4). Importantly, genes such as *CPD*, *BES1*, *HY5*, and *BBX22* are bound by SOB3 at ZT4, ZT9, and ZT24, despite being subject to AHL regulation at only one of the three time points (Fig S1). These data suggest that time point-specific regulation of target genes by AHLs is not achieved through differences in transcription factor binding. To further test this hypothesis, we examined the binding distribution of SOB3 specifically to genes identified as AHL-repressed (Fig. 10) or AHL-induced (Fig. 11) at only one or two time points. Using this approach, we again observed similar binding profiles for SOB3 at all three time points. Lastly, we examined if changes in the level of SOB3 enrichment on target genes could explain the time point-specific effects of AHLs on gene expression (Figs S2 and S3). However, we did not observe changes in SOB3 enrichment between the three time points that were likely to explain the time point-specific effects of the AHLs on the expression of target genes. This further indicates that time of day-specific effects of the AHLs on gene expression are not caused by differences in binding of these transcription factors to their target genes.

## Discussion

### AHLs regulate growth as well as the expression of specific genes throughout SD

SOB3 and other AHLs have been shown to be involved in light-mediated hypocotyl development in continuous white light and LD [1, 4–6, 8, 10]. Firstly, we showed here that SOB3 is also able to affect growth of the hypocotyl in SD (Fig. 1), as has been reported previously for AHL22 [4]. Next, we conducted RNA-seq at three different time points (ZT4, ZT9, and ZT24) to identify mis-regulated genes that may be involved in growth and development of the hypocotyl (Fig. 2). By testing three different time points throughout SD, we were hoping to uncover mis-regulation of genes that may be overlooked by only testing one time point. In fact, our data suggest that AHLs have a greater impact on gene expression during the middle of the daytime and early during the evening than at the end of the night (Fig. 2 B, C), although it is possible that there are simply fewer genes expressed in seedlings at ZT24 than at ZT4 and ZT9. Additionally, we found that the majority of genes are mis-regulated at exclusively one time point. These data suggest that AHL-regulated genes are largely affected at specific times during the day.

### AHLs regulate *PIFs* and PIF-target genes, including the newly identified *PRE1, PIL1, HFR1*, *CDF5, *and *XTR7*, in SD

In order to understand more about the specific effects SOB3 and other AHLs may be having on growth, we focused on *PIF*s and PIF-target genes. We found growth-promoting genes known to be activated by PIFs and involved in auxin biosynthesis (*YUC8*, [62]) or signaling (*SAUR*s *19/22*, [25, 26, 63, 64], and *IAA*s *19/29,* [22–24]) are repressed by AHLs at all time points (Fig. 2B). On the other hand, the *PIF*s themselves (*4*, *5*, *7*, and *8*), and *BES1*, which is involved in BR signaling [35] are only repressed at one or two time points (Fig. 2B). Notably, BES1 and PIF4 are known to function together in a complex that activates the expression of growth-promoting genes [26, 34], and our data suggest that AHLs reduce activity of this complex particularly at ZT4 by repressing the expression of both *BES1* and *PIF4* at this time point (Fig. 2B). Additionally, we identified the PIF-regulated BR biosynthesis genes *CPD* and *BR6OX2* [33, 34] as directly repressed at ZT4 (Fig. 2B, Table S4), suggesting that AHLs likely further reduce BES1-PIF4 complex activity at this time point by reducing BR biosynthesis, consequently decreasing the quantity of active BES1.

Besides *CPD* and *BR6OX2*, we have also identified five PIF target genes that are repressed and have not been previously described as repressed by AHLs: *PRE1, PIL1, HFR1*, *CDF5,* and *XTR7* (Fig. 2B). *PRE1* and *PIL1* are repressed at all time points. *PRE1* promotes PIF- and BR-mediated regulation of cell elongation [26–31]. On the other hand, PIL1, recently renamed PIF2 [65], is a PIF that functions in an atypical manner. PIL1 represses activation of gene expression, and consequently growth, by other PIF family members within a negative feedback loop [66–68]. In contrast to *PRE1* and *PIL1*, *HFR1* is only repressed at ZT9 (Fig. 2B)*. HFR1* also represses PIF-mediated activation of gene expression and growth as part of a negative feedback loop [69–72]. *CDF5* and *XTR7* are both repressed by SOB3 at ZT9 and ZT24, but not at ZT4 (Fig. 2B). CDF5 promotes the expression of some PIF-activated genes, including *YUC8* and *IAA19*, and consistently also plays a positive role in hypocotyl growth [32]. XTR7 (also known as XTH15) is a cell wall-modifying enzyme that has been shown to positively regulate petiole elongation [73]. Given the many similarities between the molecular mechanisms that regulate hypocotyl and petiole growth [21], it is likely that XTR7 also promotes hypocotyl elongation; thus, the effect of the AHLs on hypocotyl growth in SD may be caused in part by their repressive effect on *XTR7*.

Additionally, microtubules have been shown to be involved in elongation of the hypocotyl in Arabidopsis [74, 75]. In fact, several genes encoding microtubule associated proteins (MAPs) were found to be repressed by SOB3 in our RNA-seq data. For example, *MAP65-6*, whose protein localizes to the mitochondria and binds microtubules [76] was repressed at ZT4 and ZT9 and bound by SOB3 at all time points. A microtubule stabilizing gene, *WDL4* [77, 78], was repressed at ZT9 and bound by SOB3 at all time points. It fact, it was recently discovered that *WDL4* acts to modulate auxin maxima by regulating PIN trafficking during apical hook formation [79]. Therefore, our data show that SOB3 could partially control hypocotyl elongation in SD by repressing *MAP*s.

Our RNA-seq data also indicate that AHLs activate the expression of growth repressors in SD. We specifically identified growth repressors discussed in Favero et al. 21, such as *HY5, BBX21-22*, *ELF4,* and *IAA17*, as AHL-induced (Fig. 2C). However, it is interesting to note that while Favero et al. [18] observed higher expression of *BIN2* in *SOB3-D* compared to *sob3-6* using 14-day-old plants grown in LD and harvested at ZT4, we did not find *BIN2* to be differentially regulated between the two mutants at ZT4, ZT9, or ZT24. Therefore, while AHLs might inhibit growth by promoting BIN2-mediated phosphorylation and subsequently degradation of PIF3 and PIF4 [80, 81] by enhancing the expression of *BIN2* in juvenile rosettes [18], AHLs likely do not inhibit growth through this pathway in seedlings, at least those growing in SD. Given that we and the Favero et al. [18] study identified *RGA* as up-regulated in *SOB3-D* compared to *sob3-6* at ZT4 (Fig. 2C), AHLs might repress hypocotyl growth in SD partly by promoting DELLA-mediated PIF degradation and inhibition of PIF binding to target genes [82–84] specifically at this time point.

An important question is if PIF target genes are actually regulated directly by AHLs, as is suggested by both our ChIP-seq data and that from the Favero et al. [18] study. It is conceivable that the down-regulation of PIF targets that is observed in *SOB3-D* compared to *sob3-6* might simply be a downstream consequence of less PIF activity in *SOB3-D*, given that we found *PIF4*, *5*, *7*, and *8* are all AHL-repressed (Fig. 2B) and AHLs might promote DELLA-mediated degradation of PIFs, as described above. However, while these mechanisms might reduce PIF activity and consequently the activation of PIF target genes at ZT4 and ZT9, they do not appear to explain the negative effect of AHLs on the expression of these genes at ZT24. Another way the AHLs could theoretically affect PIF activity at ZT24 is by increasing *phyB* transcription, thereby enhancing phyB-mediated degradation of these transcription factors during the night, since phyB only slowly reverts back to its inactive Pr form after dusk [85–92]. However, the only photoreceptor found to be differentially regulated in our RNA-seq data was UVR8. Therefore, it seems likely that at least at ZT24 in SD, AHLs are directly repressing the expression of PIF target genes.

### SOB3: A transcription factor and a potential chromatin remodeling protein

The SOB3 binding patterns seen in our data could indicate that SOB3 is acting as a chromatin remodeling protein. The AT-hook motif was first described in the high mobility group (HMG) proteins [93–95], which are a family of non-histone chromosomal proteins found in a wide variety of eukaryotic organisms [96, 97]. HMGA proteins, similarly to SOB3, contain an AT-hook motif through which they bind AT-rich DNA [95, 98, 99]. Furthermore, HMGA proteins are also involved in the organization of chromatin [97] and the formation of the enhancesome [100, 101]. HMGA proteins compete with histone H1 for binding to linker DNA, inducing a loosening of chromatin structure [95, 102–104]. Additionally, HMGA proteins have been shown to bind to the H2A/H2B/H3/H4 nucleosome [105], indicating a role of these proteins in eviction and/or mobilization of core histones during transcriptional regulation [95]. HMGA proteins may also be involved in DNA looping and chromatin rearrangements that bring enhancesomes/promoters together so that transcription can begin [95, 100, 101]. Our SOB3 ChIP-seq binding data could indicate that SOB3 is acting similarly to HMGA proteins, where its role would be to passively regulate transcription by modifying chromatin. It is further possible that SOB3 can act as either a transcription factor or a chromatin remodeling protein depending on the specific environment in which binding occurs.

It is possible that SOB3 itself is not a chromatin remodeler, but influences chromatin remodeling through other mechanisms, such as interacting with members of chromatin remodeling complexes. These complexes are evolutionarily conserved and contain multiple subunits. Chromatin remodeling complexes can regulate chromatin structure by changing the composition of nucleosomes [106, 107]. There are four major known subfamilies of chromatin remodeling ATPases: INO80/SWR1, CHD, ISWI, and SWI/SNF [108–114]. SWR1 exchanges histone H2A for H2A.Z. This is particularly of interest, as warm temperature promotes eviction of H2A.Z from chromatin, which enhances the activation of PIF-targeted genes such as *YUC8* [18, 62, 115–119]. Additionally, there is some evidence that SOB3 represses transcription of *YUC9* by recruiting by increasing H2A.Z deposition on its promoter via recruitment of the SWR1 complex [8]. Interestingly, actin-related protein 9 (ARP9), which has been shown to be involved in SWI/SNF chromatin remodeling [120, 121], was found to be induced at all time points in our RNA-seq data. Additional experiments are needed to determine if SOB3 affects chromatin remodeling by interacting with chromatin remodeling complexes or by influencing the expression of chromatin remodeling proteins.

### Roles of AHLs in plant development: agricultural implications

Previous research has indicated that overexpression of *SOB3* and *AHL20* in Arabidopsis and Camelina delays flowering [9, 10]. Further research on newly discovered SOB3-regulated genes, such as *CDF5*, may elucidate additional mechanisms by which SOB3 is regulating flowering time. For example, loss-of-function *cdf5-1* mutants have been shown to have earlier flowering when compared to WT plants [122]. The ability to change or regulate flowering time within crop species can have many valuable advantages, such as allowing for different or increased planting seasons. Further experiments evaluating the role of *CDF5* in AHL-regulated flowering time may elucidate additional mechanisms for manipulating this advantageous agronomic trait.

## Conclusions

In summary, our data revealed that at three different time points in SD-grown seedlings, genes involved in growth of the hypocotyl are repressed, while growth repressors are induced by AHLs (Fig. 2). Additionally, we found that although somewhat different sets of genes appear to be regulated by AHLs at different time points, the binding patterns of SOB3 largely remain the same (Figs. 4, 5, 6, 10, and 11). It is important to note that we cannot rule out the possibility that some of the differences in gene expression observed between *SOB3-D* and *sob3-6* in our RNA-seq experiment are caused indirectly by the phenotypic difference in hypocotyl length between the two genotypes, and thus are not directly regulated by AHLs at the time points used in this experiment. However, we suspect that at least some of the time point-specific effects of the AHLs on gene expression identified in this experiment are attributable to the composition of AHL-containing protein complexes differing at ZT4, ZT9, and ZT24. The activities of AHL complexes are perhaps modified through interactions with different non-AHL transcription factors that are important for regulating gene expression and ultimately hypocotyl growth at specific time points in SD. Alternatively, changes in the specific AHL family members present in AHL complexes at the different time points might explain the different effects of these transcription factors on gene expression at the three time points. It is also possible that AHLs regulates gene expression in SD by acting as a chromatin remodeling proteins, since the AT-hook motif is also found in the chromatin remodeling HMGA proteins. Alternatively, rather than AHLs acting as chromatin remodeler themselves in SD, they might engage members of chromatin remodeling complexes to regulate gene expression. Future studies should focus on testing these hypotheses, in order to clarify the precise molecular mechanisms that govern regulation of gene expression and hypocotyl growth by SOB3 and other AHLs.

## Methods

The aim of this study was to undercover unique molecular events that promote growth at different time points during SD conditions in Arabidopsis. More precisely, we aimed to identify specific genes that are bound by SOB3 and are mis-regulated between *sob3* mutants that may be involved in growth and development of the hypocotyl in Arabidopsis. The overall design contains two main experiments: ChIP-seq and RNA-seq, which are described in detail below.

### Plant materials and growth conditions

The Arabidopsis (*Arabidopsis* thaliana) lines used in the study are all in the Columbia (Col-0) background and have been described previously: *SOB3-D* and *sob3-6* [1]; *ProSOB3::SOB3-GFP sob3-4* [5]. Seeds had been surface-sterilized and were grown on full-strength MS medium containing 0.6% Gelzan CM (Sigma-Aldrich) and 1% sucrose (w/v). Seeds were stratified in the dark at 4 °C for 3 days. Plants were grown in an MLR-351 plant growth chamber. The chamber was kept at a temperature of 22 °C and a short-day photoperiod (8 h light/16 h dark) for five-six days. 3LS was used as the light setting during the daytime, producing white fluorescent light with a fluence rate of approximately 35–55 μmol/m^2^/sec. For the RNA-seq and ChIP-seq experiments, seedlings were harvested at one of three time points, 4 h after dawn (ZT4), 1 h after lights off (ZT9), or at dawn the next day (ZT24), and immediately frozen in liquid nitrogen.

Arabidopsis Locus Identifiers:

- PIF4: AT2G43010
- CPD: AT5G05690
- BES1: AT1G19350
- SAUR24: AT5G18080
- BR6OX2: AT3G30180
- PRE1: AT5G39860
- SAUR19: AT5G18010
- SAUR22: AT5G18050
- YUC8: AT4G28720
- PIL1: AT2G46970
- IAA19: AT3G15540
- IAA29: AT4G32280
- HRF1: AT1G02340
- PIF5: AT3G59060
- PIF7: AT5G61270
- PIF8: AT4G00050
- CDF5: AT1G69570
- XTR7: AT4G14130
- ELF4: AT2G40080
- HY5: AT5G11260
- BBX22: AT1G78600
- RGA: AT2G01570
- PRR9: AT2G46790
- IAA17: AT1G04250
- ELF3: AT2G25930
- BBX21: AT1G75540

### Hypocotyl measurements

Seedlings used for quantification of the *sob3* mutant phenotypes were grown in the same conditions as described above. WT seedings are of *Arabidopsis thaliana* (L.) Col-0 ecotype. Five-day-old seedings were transferred to transparencies, which were digitized with a flatbed scanner. The transparencies included a ruler for measuring a 1 mm length to set the parameters for measurements in ImageJ (NIH). hypocotyls were measured from the top of hypocotyl to the beginning of the roots using the segmented line tool. The measurements were transferred to an Excel spreadsheet for analysis. A two-tailed Welch's t-test was used to check for significant differences in hypocotyl length between the mutants and the wild type.

### RNA sequencing (RNA-seq) and data analysis

20–35 whole seedlings were harvested in triplicate at the indicated time points, with plants for different biological replicates grown on different plates. Samples for different genotypes within the same biological replicate set were harvested from the same plate(s). This experimental design was used to minimize the impact of plate-to-plate variation on gene expression. Total RNA was extracted using the RNeasy Plant Mini Kit (QIAGEN) according to manufacturer’s instructions, including on-column DNase digestion to eliminate genomic DNA. Isolated RNA was subjected to library preparation using the KAPA Stranded mRNA-Seq Kit (Kapa Biosystems) with NEBNext Multiplex Oligos for Illumina (New England Biolabs) used as adapters and Agencourt AMPure XP (Beckman Coulter) beads used instead of KAPA Pure Beads. Libraries were pooled and 84- or 86-bp, single-read sequences were obtained with an Illumina NextSeq500 sequencer. Raw data files (bcl format) were converted to fastq files by bcl2fastq (Illumina). Over 50% of reads were mapped to the Arabidopsis TAIR10 cDNA reference using Bowtie [123] with the following parameters: ‘–all –best –strata’. The total number of mapped reads per sample was 6–12 million. Differentially expressed transcripts between pairs of samples were identified using the edgeR package in R/Bioconductor [124] with an FDR cutoff of 0.05. AHL-regulated genes were defined as nuclear-encoded genes for which at least one transcript was differentially regulated between sob3-6 and SOB3-D. Venn diagrams were generated using an online tool available at http://bioinformatics.psb.ugent.be/webtools/Venn/.

### Chromatin Immunoprecipitation sequencing (ChIP-seq)

The ChIP procedure included three biological replicates for each time point. Chromatin immunoprecipitation for ChIP-seq was performed essentially as described previously [18]. 1.5 g of *ProSOB3::SOB3-GFP sob3-4* [5] whole seedlings were harvested at ZT4 and immediately frozen using liquid nitrogen. Samples were ground to a fine powder using an MB1200 Multi-beads Shocker (Yasui Kikai) and cross-linking performed in 1% formaldehyde for 10 min, after which the nuclear fraction was isolated. Chromatin suspended in SDS lysis buffer (50 mm Tris–HCl [pH 7.8], 1% SDS, 10 mM EDTA) was diluted approximately five-fold with ChIP dilution buffer (50 mm Tris–HCl [pH 7.8], 0.167 M NaCl, 1.1% Triton X-100, 0.11% sodium deoxycholate, 1 Roche cOmplete EDTA-free tablet/50 mL solution) and sheared at 5 °C for 15 min using a Covaris S2 Focused-ultrasonicator with milliTUBE 1 mL AFA Fiber tubes (Covaris) and the following settings: duty cycle 5%, intensity 4, and cycles per burst 200. This produced an average fragment size of approximately 100–300 bp. Sonicated chromatin was immunoprecipitated using an anti-GFP antibody (ab290, Abcam) and Dynabeads Protein A (Thermo Fisher Scientific) at 4C. Following incubation with antibody, samples were washed once with low salt RIPA buffer (50mMTris-HCl [pH 7.8], 150mMNaCl, 1mMEDTA, 0.1% SDS, 1% Triton X-100, 0.1% sodium deoxycholate, 1 Roche cOmplete Ultra tablet/50 mL solution), twice with high salt RIPA buffer (50mMTris-HCl [pH 7.8], 500mMNaCl, 1mMEDTA, 0.1% SDS, 1% Triton X-100, 0.1% sodium deoxycholate, 1 Roche cOmplete Ultra tablet/50 mL solution), once with LNDET (10 mM Tris–HCl [pH 7.8], 250 mM LiCl, 1% IGEPAL CA-630, 1% sodium deoxycholate, 1 mM EDTA) and once with TE buffer. Chromatin was eluted from Dynabeads and cross-linking reversed at 65 °C in a solution containing 10 mM Tris–HCl [pH 7.8], 300 mM NaCl, 5 mM EDTA and 0.5% SDS. Phenol–chloroform extraction followed by ethanol precipitation was used to isolate DNA. Sequencing libraries were prepared essentially as described in Rymen et al*.* (2019). Isolated DNA was quantitated using the Qubit dsDNA HS Assay Kit with the Qubit 2.0 Fluorometer (Thermo Fisher Scientific), and 3.65 ng of ChIPed or input (for control libraries) DNA was subjected to library preparation. Libraries were prepared using the KAPA Hyper Prep Kit for Illumina (KAPA Biosystems) and Illumina compatible adaptors (New England Biolabs). Libraries were pooled and 86-bp, single-read sequences were obtained with an Illumina NextSeq500 sequencer.

### Chromatin Immunoprecipitation sequencing (ChIP-seq) data analysis

Raw data files (bcl format) were converted to fastq files by bcl2fastq (Illumina). Over 70% of reads were uniquely mapped to the Arabidopsis TAIR10 reference using Bowtie [123] with the setting ‘‘-m 1.’’ The total number of uniquely mapped reads per sample was 11–26 million. Peaks were called by comparing ChIP samples with the input using the ‘‘callpeak’’ command in MACS2 [125]. Fold-enrichment bdg format peak files were generated by using the treatment pileup and control lambda output files generated from ‘‘callpeak’’ as inputs for the MACS2 ‘‘bdgcmp’’ command with the setting ‘‘-m FE.’’ These fold-enrichment bdg files were then converted to bigWig files, which were in turn used to generate the figures showing SOB3 binding distribution or relative binding on target genes via the computeMatrix and plotHeatmap or plotProfile commands, respectively, in deepTools [126]. Peaks were visualized using the Integrative Genomics Viewer (IGV) version 2.3.88 [127, 128]. Motif analysis was performed using MEME-ChIP [129] with Arabidopsis PBM motifs selected as known motifs [56]. 300 bp nucleotide sequences centered at the SOB3-GFP ChIP-seq peak summits were used as primary input sequences for MEME-ChIP analysis. Peaks were annotated using HOMER [130]. Venn diagrams were generated using an online tool available at http://bioinformatics.psb.ugent.be/webtools/Venn/.

### Quantification and statistical analysis

Where indicated, a two-tailed Welch’s t test was performed where *P* ≤ 0.05 = *; *P* ≤ 0.005 = **; *P* ≤ 0.0005 = ***; and *P* ≤ 0.00005 = ****

Gene ontology enrichment analysis was performed via BINGO [131]. GO term results were viewed and heatmaps were created using MeV 4.8.1 [132].

## Supplementary Information

**Additional file 1:** **Supplementary Table 1.** CPM values and edgeR results (presented for each transcript) for RNA-seq data generated from sob3-6 and SOB3-D seedlings harvested at ZT4 (A), ZT9 (B), and ZT24 (C).

**Additional file 2: Supplementary Table 2.** The fold change of PIF-target genes from Figure 2. Logfc indicates log2 difference in gene expression between sob3-6 and SOB3-D seedlings. Highlighted values denote transcripts that are differentially regulated between the two genotypes for the given time point as indicated by a p-value (FDR-adjusted *p*-value) < 0.05

**Additional file 3: Supplementary Table 3.** Peaks identified from SOB3-GFP ChIP-seq data, using MACS2, for seedlings harvested at ZT4 (A-C), ZT9 (D-F), and ZT24 (G-I). Data from three different biological replicates for each time point are presented individually.

**Additional file 4: Supplementary Table 4.** Binding of SOB3, based on the ChIP-seq data, to AHL-repressed or AHL-induced genes named in Fig. 2B or 2C, respectively. * = bound by SOB3 in one rep; ** = bound by SOB3 in two reps; *** = bound by SOB3 in three reps. Blue highlighting indicates the gene is counted as a SOB3-bound gene at that time point (i.e. it was bound in at least 2 ChIP-seq reps). “binds uORF” indicates that although annotation using HOMER did not detect the gene as bound by SOB3 (i.e. the gene’s TSS was not the closest TSS to any SOB3 ChIP-seq peak), a uORF located just upstream of the gene was identified as SOB3-bound.

**Additional file 5: Supplementary Figure 1.** Binding of SOB3 to loci associated with BES1 (A), CPD (B), BBX22 (C), or PIF4 (D) based on the ChIP-seq data generated from ProSOB3::SOB3-GFP sob3-4 seedlings harvested at ZT4, ZT9, or ZT24. Plots show relative fold enrichment in the ChIP samples compared to their respective input controls.

**Additional file 6: Supplementary Figure 2.** Relative binding of SOB3, based on the ChIP-seq data generated from ProSOB3::SOB3-GFP sob3-4 seedlings harvested at ZT4, ZT9, or ZT24, to genes identified as induced or repressed by AHLs at only a single time point from the RNA-seq data for SOB3-D and sob3-6. (A) Relative binding of SOB3 to genes identified as repressed (left) or induced (right) by AHLs only at ZT4. (B) Relative binding of SOB3 to genes identified as repressed (left) or induced (right) by AHLs only at ZT9. (C) Relative binding of SOB3 to genes identified as repressed (left) or induced (right) by AHLs only at ZT24.

**Additional file 7: Supplementary Figure 3.** Relative binding of SOB3, based on the ChIP-seq data generated from ProSOB3::SOB3-GFP sob3-4 seedlings harvested at ZT4, ZT9, or ZT24, to genes identified as induced or repressed by AHLs at only two time points from the RNA-seq data for SOB3-D and sob3-6. (A) Relative binding of SOB3 to genes identified as repressed (left) or induced (right) by AHLs at ZT4 and ZT9 but not at ZT24. (B) Relative binding of SOB3 to genes identified as repressed (left) or induced (right) by AHLs at ZT4 and ZT24 but not at ZT9. (C) Relative binding of SOB3 to genes identified as repressed (left) or induced (right) by AHLs at ZT9 and ZT24 but not at ZT4.

## Data Availability

The datasets generated and/or analyzed during the current study are available in the Gene Expression Omnibus repository, https://www.ncbi.nlm.nih.gov/geo/query/acc.cgi?acc=GSE189265.

## References

[1] Street IH, Shah PK, Smith AM, Avery N, Neff MM. The AT-hook-containing proteins SOB3/AHL29 and ESC/AHL27 are negative modulators of hypocotyl growth in Arabidopsis. Plant J. 2008;54(1):1–14. 10.1111/j.1365-313x.2007.03393.x. 10.1111/j.1365-313X.2007.03393.x 18088311

[2] Zhou L, Liu Z, Liu Y, Kong D, Li T, Yu S, Mei H, Xu X, Liu H, Chen L, Luo L. A novel gene OsAHL1 improves both drought avoidance and drought tolerance in rice. Sci Rep. 2006;6:30264. 10.1038/srep30264 PMC4958981 27453463

[3] Wong MM, Bhaskara GB, Wen TN, Lin WD, Nguyen TT, Chong GL, Verslues PE. Phosphoproteomics of Arabidopsis highly ABA-Induced1 identifies AT-Hook-Like10 phosphorylation required for stress growth regulation. Proc Natl Acad Sci USA. 2019;116:2354–63. 10.1073/pnas.1819971116. 10.1073/pnas.1819971116 PMC6369736 30670655

[4] Xiao C, Chen F, Yu X, Lin C, Fu YF. Over-expression of an AT-hook gene, AHL22, delays flowering and inhibits the elongation of the hypocotyl in Arabidopsis thaliana. Plant Mol Biol. 2009;71:39–50. 10.1007/s11103-009-9507-9. 10.1007/s11103-009-9507-9 19517252

[5] Favero DS, Jacques CN, Iwase A, Le KN, Zhao J, Sugimoto K, Neff MM. SUPPRESSOR OF PHYTOCHROME B4-#3 Represses Genes Associated with Auxin Signaling to Modulate Hypocotyl Growth. Plant Physiol. 2016;171(4):2701–16. 10.1104/pp.16.00405. 10.1104/pp.16.00405 PMC4972272 27342309

[6] Zhao J, Favero DS, Peng H, Neff MM. Arabidopsis thaliana AHL family modulates hypocotyl growth redundantly by interacting with each other via the PPC/DUF296 domain. Proc Natl Acad Sci USA. 2013;110(48). 10.1073/pnas.1219277110 10.1073/pnas.1219277110 PMC3845178 24218605

[7] Peng H, Zhao J, Neff MM. ATAF2 integrates Arabidopsis brassinosteroid inactivation and seedling photomorphogenesis. Development. 2015;142(23):4129–38. 10.1242/dev.124347. 10.1242/dev.124347 26493403

[8] Lee K, Seo, PJ Coordination of matrix attachment and ATP-dependent chromatin remodeling regulate auxin biosynthesis and Arabidopsis hypocotyl elongation. Plos One. 2017;12(7). 10.1371/journal.pone.0181804 10.1371/journal.pone.0181804 PMC5529009 28746399

[9] Tayengwa R, Koirala PS, Pierce CF, Werner BE, Neff MM. Overexpression of AtAHL20 causes delayed flowering in Arabidopsis via repression of FT expression. BMC Plant Biol. 2020;20:559. 10.1186/s12870-020-02733-5. 10.1186/s12870-020-02733-5 PMC7731500 33308168

[10] Koirala PS, Neff MM. Improving seed size, seed weight and seedling emergence in Camelina sativa by overexpressing the Atsob3-6 gene variant. Transgenic Res. 2020;29(4):409–18. 10.1007/s11248-020-00208-9. 10.1007/s11248-020-00208-9 32748170

[11] Elich TD, Chory J. Biochemical Characterization of Arabidopsis Wild-Type and Mutant Phytochrome B Holoproteins. Plant Cell. 1997;9(12):2271. 10.2307/3870584. 10.1105/tpc.9.12.2271 PMC157073 9437866

[12] Weigel D, Ahn JH, Blázquez MA, Borevitz JO, Christensen SK, Fankhauser C, Ferrandiz C, Kardailsky I, Malancharuvil EJ, Neff, MM, Nguyen JT, Sato S, Wang ZY, Xia Y, Dixon RA, Harrison MJ, Lamb CJ, Yanofsky MF, Chory J. Activation Tagging in Arabidopsis. Plant Physiol. 2000;122(4):1003–14. 10.1104/pp.122.4.1003. 10.1104/pp.122.4.1003 PMC1539247 10759496

[13] Neff MM, Fankhauser C, Chory J. Light: an indicator of time and place. Genes Dev. 2000;14:257–71. 10673498

[14] Fujimoto S, Matsunaga S, Yonemura M, Uchiyama S, Azuma T, Fukui K. Identification of a novel plant MAR DNA binding protein localized on chromosomal surfaces. Plant Mol Biol. 2004;56(2):225–39. 10.1007/s11103-004-3249-5. 10.1007/s11103-004-3249-5 15604740

[15] Zhao J, Favero DS, Qiu J, Roalson EH, Neff MM. Insights into the evolution and diversification of the AT-hook Motif Nuclear Localized gene family in land plants. BMC Plant Biol. 2014;14(266):11. 10.1186/s12870-014-0266-7 PMC4209074 25311531

[16] Lim PO, Kim Y, Breeze E, Koo JC, Woo HR, Ryu JS, Park DH, Beynon J, Tabrett A, Buchanan-Wollaston V, Nam HG. Overexpression of a chromatin architecture-controlling AT-hook protein extends leaf longevity and increases the post-harvest storage life of plants. Plant J. 2007;52(6):1140–53. 10.1111/j.1365-313x.2007.03317.x. 10.1111/j.1365-313X.2007.03317.x 17971039

[17] Jia QS, Zhu J, Xu XF, Lou Y, Zhang ZL, Zhang ZP, Yang ZN. Arabidopsis AT-hook Protein TEK Positively Regulates the Expression of Arabinogalactan Proteins for Nexine Formation. Mol Plant. 2015;8(2):251–60. 10.1016/j.molp.2014.10.001. 10.1016/j.molp.2014.10.001 25616387

[18] Favero DS, Kawamura A, Shibata M, Takebayashi A, Jung JH, Suzuki T, Jaeger KE, Ishida T, Iwase A, Wigge PA, Neff MM, Sugimoto K. AT-Hook Transcription Factors Restrict Petiole Growth by Antagonizing PIFs. Curr Biol. 2020;30(8). 10.1016/j.cub.2020.02.017 10.1016/j.cub.2020.02.017 32197081

[19] Nozue K, Covington MF, Duek PD, Lorrain S, Fankhauser C, Harmer SL, Maloof JN. Rhythmic growth explained by coincidence between internal and external cues. Nature. 2007;448(7151):358–61. 10.1038/nature05946 17589502

[20] Box MS, Huang BE, Domijan M, Jaeger KE, Khattak AK, Yoo SJ, Sedivy EL, Jones DM, Hearn TJ, Webb AA, Grant A, Locke JC, Wigge PA. ELF3 controls thermoresponsive growth in Arabidopsis. Curr Biol. 2015;25(2):194–9. 10.1016/j.cub.2014.10.076 25557663

[21] Favero DS, Lambolez A, Sugimoto K. Molecular pathways regulating elongation of aerial plant organs: A focus on light, the circadian clock, and temperature. Plant J. 2021;105(2):392–420. 10.1111/tpj.14996. 10.1111/tpj.14996 32986276

[22] Kunihiro A, Yamashino T, Nakamichi N, Niwa Y, Nakanishi H, Mizuno T. Phytochrome-interacting factor 4 and 5 (PIF4 and PIF5) activate the homeobox ATHB2 and auxin-inducible IAA29 genes in the coincidence mechanism underlying photoperiodic control of plant growth of Arabidopsis thaliana. Plant Cell Physiol. 2011;52:1315–29. 10.1093/pcp/pcr076 21666227

[23] Sun J, Qi L, Li Y, Zhai Q, Li C. PIF4 and PIF5 transcription factors link blue light and auxin to regulate the phototropic response in Arabidopsis. Plant Cell, 2013;25:2102–14. 10.1105/tpc.113.112417 PMC3723615 23757399

[24] Pucciariello O, Legris M, Rojas CC, Iglesias MJ, Hernando CE, Dezar C, Vazquez M, Yanofsky MJ, Finlayson SA, Prat S, Casal JJ. Rewiring of auxin signaling under persistent shade. Proc Natl Acad Sci. 2018;115(21):5612–7. 10.1073/pnas.1721110115. 10.1073/pnas.1721110115 PMC6003476 29724856

[25] Hornitschek P, Kohnen MV, Lorrain S, Rougemont J, Ljung K, Lopez-Vidriero I, Franco-Zorrilla JM, Solano R, Trevisan M, Pradervand S, Xenarios I, Fankhauser C. Phytochrome interacting factors 4 and 5 control seedling growth in changing light conditions by directly controlling auxin signaling. Plant J. 2012;71:699–711. 10.1111/j.1365-313X.2012.05033.x 22536829

[26] Oh E, Zhu JY, Wang ZY. Interaction between BZR1 and PIF4 integrates brassinosteroid and environmental responses. Nat Cell Biol. 2012;14:802–9. 10.1038/ncb2545 PMC3703456 22820378

[27] Lee S, Lee S, Yang KY, Kim YM, Park SY, Kim SY, Soh MS. Overexpression of PRE1 and its homologous genes activates Gibberellin-dependent responses in Arabidopsis thaliana. Plant Cell Physiol. 2006;47:591–600. 10.1093/pcp/pcj026 16527868

[28] Bai MY, Fan M, Oh E, Wang ZY. A triple helix-loop-helix/basic helix-loop-helix cascade controls cell elongation downstream of multiple hormonal and environmental signaling pathways in Arabidopsis. Plant Cell, 2012;24:4917–29. 10.1105/tpc.112.105163 PMC3556966 23221598

[29] Bai MY, Shang JX, Oh E, Fan M, Bai Y, Zentella R, Sun TP, Wang ZY. Brassinosteroid, gibberellin and phytochrome impinge on a common transcription module in Arabidopsis. Nat Cell Biol. 2012;14:810–7. 10.1038/ncb2546 PMC3606816 22820377

[30] Hao Y, Oh E, Choi G, Liang Z, Wang Z. Interactions between HLH and bHLH Factors Modulate Light-Regulated Plant Development. Mol Plant. 2012;5(3):688–97. 10.1093/mp/sss011. 10.1093/mp/sss011 PMC3628346 22331621

[31] Ikeda M, Fujiwara S, Mitsuda N, Ohme-Takagi M. A triantagonistic basic helix-loop-helix system regulates cell elongation in Arabidopsis. Plant Cell, 2012;24:4483–97. 10.1105/tpc.112.105023 PMC3531847 23161888

[32] Martin G, Rovira A, Veciana N, Soy J, Toledo-Ortiz G, Gommers CMM, Boix M, Henriques R, Minguet EG, Alabadi D, Halliday KJ, Leivar P, Monte E. Circadian Waves of Transcriptional Repression Shape PIF-Regulated Photoperiod-Responsive Growth in Arabidopsis. Curr Biol. 2018;28(2):311–8. 10.1016/j.cub.2017.12.021. 10.1016/j.cub.2017.12.021 29337078

[33] Wei Z, Yuan T, Tarkowská D, Kim J, Nam HG, Novák O, He K, Gou XP, Li J. Brassinosteroid Biosynthesis Is Modulated via a Transcription Factor Cascade of COG1, PIF4, and PIF5. Plant Physiol. 2017;174(2):1260–73. 10.1104/pp.16.01778. 10.1104/pp.16.01778 PMC5462011 28438793

[34] Martínez C, Espinosa‐Ruíz A, de Lucas M, Bernardo‐García S, Franco‐Zorrilla JM, Prat S. PIF4‐induced BR synthesis is critical to diurnal and thermomorphogenic growth. The EMBO Journal, 2018;37(23). doi:10.15252/embj.201899552 10.15252/embj.201899552 PMC6276883 30389669

[35] Yin Y, Vafeados D, Tao Y, Yoshida S, Asami T, Chory J. A New Class of Transcription Factors Mediates Brassinosteroid-Regulated Gene Expression in Arabidopsis. Cell. 2005;120(2):249–59. 10.1016/j.cell.2004.11.044. 10.1016/j.cell.2004.11.044 15680330

[36] Saito M, Kondo Y, Fukuda H. BES1 and BZR1 Redundantly Promote Phloem and Xylem Differentiation. Plant Cell Physiol. 2018;59(3):590–600. 10.1093/pcp/pcy012. 10.1093/pcp/pcy012 29385529

[37] Chang CJ, Maloof JN, Wu S. COP1-Mediated Degradation of BBX22/LZF1 Optimizes Seedling Development in Arabidopsis. Plant Physiol. 2011;156(1):228–39. 10.1104/pp.111.175042. 10.1104/pp.111.175042 PMC3091042 21427283

[38] Osterlund MT, Hardtke CS, Wei N, Deng XW. Targeted destabilization of HY5 during light-regulated development of Arabidopsis. Nature, 2000;405:462–6. 10.1038/35013076. 10.1038/35013076 10839542

[39] Ang LH, Chattopadhyay S, Wei N, Oyama T, Okada K, Batschauer A, Deng XW. Molecular interaction between COP1 and HY5 defines a regulatory switch for light control of Arabidopsis development. Mol Cell. 1998;1:213–22. 10.1016/s1097-2765(00)80022-2. 10.1016/s1097-2765(00)80022-2 9659918

[40] Burko Y, Seluzicki A, Zander M, Pedmale UV, Ecker JR, Chory J. Chimeric activators and repressors define HY5 activity and reveal a light-regulated feedback mechanism. Plant Cell. 2020;32:967–83. 10.1105/tpc.19.00772. 10.1105/tpc.19.00772 PMC7145465 32086365

[41] Bursch K, Toledo-Ortiz G, Pireyre M, Lohr M, Braatz C, Johansson H. Identification of BBX proteins as rate-limiting cofactors of HY5. Nature Plants. 2020;6(8):921–8. 10.1038/s41477-020-0725-0. 10.1038/s41477-020-0725-0 32661279

[42] Xu D, Jiang Y, Li J, Lin F, Holm M, Deng XW. BBX21, an Arabidopsis B-box protein, directly activates HY5 and is targeted by COP1 for 26S proteasome-mediated degradation. Proc Natl Acad Sci USA. 2016;113:7655–60. 10.1073/pnas.1607687113 PMC4941485 27325768

[43] Gallavotti A, Malcomber S, Gaines C, Stanfield S, Whipple C, Kellogg E, Schmidt RJ. BARREN STALK FASTIGIATE1 Is an AT-Hook Protein Required for the Formation of Maize Ears. Plant Cell, 2011;23(5):1756–71. 10.1105/tpc.111.084590. 10.1105/tpc.111.084590 PMC3123940 21540434

[44] Jin Y, Luo Q, Tong H, Wang A, Cheng Z, Tang J, Li D, Zhao X, Li X, Wan J, Jiao Y, Chu C, Zhu L. An AT-hook gene is required for palea formation and floral organ number control in rice. Dev Biol. 2011;359(2):277–88. 10.1016/j.ydbio.2011.08.023. 10.1016/j.ydbio.2011.08.023 21924254

[45] Lou Y, Xu X, Zhu J, Gu J, Blackmore S, Yang Z. The tapetal AHL family protein TEK determines nexine formation in the pollen wall. Nature Communications, 2014;5(1). doi:10.1038/ncomms4855 10.1038/ncomms4855 PMC4024750 24804694

[46] Matsushita A, Furumoto T, Ishida S, Takahashi Y. AGF1, an AT-Hook Protein, Is Necessary for the Negative Feedback of AtGA3ox1 Encoding GA 3-Oxidase. Plant Physiol. 2007;143(3):1152–62. 10.1104/pp.106.093542. 10.1104/pp.106.093542 PMC1820926 17277098

[47] Vom Endt D, Soares e Silva M, Kijne JW, Pasquali G, Memelink J. Identification of a bipartite jasmonate-responsive promoter element in the Catharanthus roseus ORCA3 transcription factor gene that interacts specifically with AT-Hook DNA-binding proteins. Plant Physiol. 2007;144(3):1680–9. 10.1104/pp.107.096115 PMC1914126 17496112

[48] Ng K, Yu H, Ito T. AGAMOUS Controls GIANT KILLER, a Multifunctional Chromatin Modifier in Reproductive Organ Patterning and Differentiation. PLoS Biology, 2009;7(11). doi:10.1371/journal.pbio.1000251 10.1371/journal.pbio.1000251 PMC2774341 19956801

[49] Ng K, Ito T. Shedding light on the role of AT-hook/PPC domain protein in Arabidopsis thaliana. Plant Signal Behav. 2010;5(2):200–1. 10.4161/psb.5.2.11111. 10.4161/psb.5.2.11111 PMC2884135 20173412

[50] Xu Y, Gan E, Ito T. The AT-hook/PPC domain protein TEK negatively regulates floral repressors including MAF4 and MAF5. Plant Signal Behav. 2013;8(8). doi:10.4161/psb.25006 10.4161/psb.25006 PMC3999084 23733063

[51] Yun J, Kim YS, Jung JH, Seo PJ, Park CM. The AT-hook Motif-containing Protein AHL22 Regulates Flowering Initiation by Modifying FLOWERING LOCUS T Chromatin in Arabidopsis. J Biol Chem. 2012;287(19):15307–16. 10.1074/jbc.m111.318477. 10.1074/jbc.M111.318477 PMC3346147 22442143

[52] Bailey TL, Johnson J, Grant CE, Noble WS. The MEME Suite. Nucleic Acids Research, 2015;43(W1):W39–W49. 10.1093/nar/gkv416 PMC4489269 25953851

[53] Kosugi S, Ohashi Y (2002). DNA binding and dimerization specificity and potential targets for the TCP protein family. Plant J.

[54] Gupta S, Stamatoyannopoulos JA, Bailey TL, Noble WS. Quantifying similarity between motifs. Genome Biol. 2007;8:R24. 10.1186/gb-2007-8-2-r24 PMC1852410 17324271

[55] Schommer C, Palatnik JF, Aggarwal P, Chetelat A, Cubas P, Farmer EE, Nath U, Weigel D (2008). Control of jasmonate biosynthesis and senescence by miR319 targets. PLoS Biol.

[56] Franco-Zorrilla JM, Lopez-Vidriero I, Carrasco JL, Godoy M, Vera P, Solano R (2014). DNA-binding specificities of plant transcription factors and their potential to define target genes. Proc Natl Acad Sci U S A.

[57] Yin D, Liu X, Shi Z, Li D, Zhu L (2018). An AT-hook protein DEPRESSED PALEA1 physically interacts with the TCP Family transcription factor RETARDED PALEA1 in rice. Biochem Biophys Res Commun.

[58] Oh E, Yamaguchi S, Hu J, Yusuke J, Jung B, Paik I, Lee HS, Sun TP, Kamiya Y, Choi G. PIL5, a phytochrome-interacting bHLH protein, regulates gibberellin responsiveness by binding directly to the GAI and RGA promoters in Arabidopsis seeds. Plant Cell. 2007;19:1192–208. 10.1105/tpc.107.050153 PMC1913757 17449805

[59] Kim DH, Yamaguchi S, Lim S, Oh E, Park J, Hanada A, Kamiya Y, Choi G (2008). SOMNUS, a CCCH-type zinc finger protein in Arabidopsis, negatively regulates light-dependent seed germination downstream of PIL5. Plant Cell.

[60] Zhang Y, Mayba O, Pfeiffer A, Shi H, Tepperman JM, Speed TP, Quail PH (2013). A quartet of PIF bHLH factors provides a transcriptionally centered signaling hub that regulates seedling morphogenesis through differential expression-patterning of shared target genes in Arabidopsis. PLoS Genet.

[61] Huth JR, Bewley CA, Nissen MS, Evans JN, Reeves R, Gronenborn AM, Clore GM (1997). The solution structure of an HMG-I(Y)-DNA complex defines a new architectural minor groove binding motif. Nat Struct Biol.

[62] Sun J, Qi L, Li Y, Chu J, Li C (2012). PIF4-mediated activation of YUCCA8 expression integrates temperature into the auxin pathway in regulating arabidopsis hypocotyl growth. PLoS Genet.

[63] Spartz AK, Lee SH, Wenger JP, Gonzalez N, Itoh H, Inze D, Peer WA, Murphy AS, Overvoorde PJ, Gray WM (2012). The SAUR19 subfamily of SMALL AUXIN UP RNA genes promote cell expansion. Plant J.

[64] Spartz AK, Ren H, Park MY, Grandt KN, Lee SH, Murphy AS, Sussman MR, Overvoorde PJ, Gray WM. SAUR Inhibition of PP2C-D Phosphatases Activates Plasma Membrane H+-ATPases to Promote Cell Expansion in Arabidopsis. Plant Cell. 2014;26:2129–42. 10.1105/tpc.114.126037 PMC4079373 24858935

[65] Pham VN, Kathare PK, Huq E (2018). Phytochromes and Phytochrome Interacting Factors. Plant Physiol.

[66] Roig-Villanova I, Bou J, Sorin C, Devlin PF, Martinez-Garcia JF (2006). Identification of primary target genes of phytochrome signaling. Early transcriptional control during shade avoidance responses in Arabidopsis. Plant Physiol.

[67] Li L, Zhang Q, Pedmale UV, Nito K, Fu W, Lin L, Hazen SP, Chory J (2014). PIL1 participates in a negative feedback loop that regulates its own gene expression in response to shade. Mol Plant.

[68] Luo Q, Lian HL, He SB, Li L, Jia KP, Yang HQ. COP1 and phyB Physically Interact with PIL1 to Regulate Its Stability and Photomorphogenic Development in Arabidopsis. Plant Cell. 2014;26:2441–56. 10.1105/tpc.113.121657 PMC4114944 24951480

[69] Fairchild CD, Schumaker MA, Quail PH (2000). HFR1 encodes an atypical bHLH protein that acts in phytochrome A signal transduction. Genes Dev.

[70] Fankhauser C, Chory J (2000). RSF1, an Arabidopsis locus implicated in phytochrome A signaling. Plant Physiol.

[71] Duek PD, Fankhauser C (2003). HFR1, a putative bHLH transcription factor, mediates both phytochrome A and cryptochrome signalling. Plant J.

[72] Hornitschek P, Lorrain S, Zoete V, Michielin O, Fankhauser C (2009). Inhibition of the shade avoidance response by formation of non-DNA binding bHLH heterodimers. EMBO J.

[73] Sasidharan R, Chinnappa CC, Staal M, Elzenga JT, Yokoyama R, Nishitani K, Voesenek LA, Pierik R (2010). Light quality-mediated petiole elongation in Arabidopsis during shade avoidance involves cell wall modification by xyloglucan endotransglucosylase/hydrolases. Plant Physiol.

[74] Le J, Vandenbussche F, De Cnodder T, Van Der Straeten D, Verbelen J (2005). Cell elongation and microtubule behavior in the Arabidopsis hypocotyl: Responses to ethylene and Auxin. J Plant Growth Regul.

[75] Crowell EF, Timpano H, Desprez T, Franssen-Verheijen T, Emons A, Höfte H, Vernhettes S. Differential regulation of cellulose orientation at the inner and outer face of epidermal cells in the Arabidopsis hypocotyl. Plant Cell. 2011;23(7):2592–605. 10.1105/tpc.111.087338. 10.1105/tpc.111.087338 PMC3226210 21742992

[76] Mao T, Jin L, Li H, Liu B, Yuan M (2005). Two Microtubule-Associated Proteins of the Arabidopsis MAP65 Family Function Differently on Microtubules. Plant Physiol.

[77] Yuen CY, Pearlman RS, Silo-Suh L, Hilson P, Carroll KL, Masson PH (2003). WVD2 and WDL1 modulate helical organ growth and anisotropic cell expansion in Arabidopsis. Plant Physiol.

[78] Perrin RM, Wang Y, Yuen CY, Will J, Masson PH (2007). WVD2 is a novel microtubule-associated protein in Arabidopsis thaliana. Plant J.

[79] Deng J, Wang X, Liu Z, Mao T. The microtubule-associated protein WDL4 modulates auxin distribution to promote apical hook opening in Arabidopsis. Plant Cell. 2021;33(6):1927–44. 10.1093/plcell/koab080. 10.1093/plcell/koab080 PMC8290285 33730147

[80] Bernardo-Garcia S, de Lucas M, Martinez C, Espinosa-Ruiz A, Daviere JM, Prat S (2014). BR-dependent phosphorylation modulates PIF4 transcriptional activity and shapes diurnal hypocotyl growth. Genes Dev.

[81] Ling JJ, Li J, Zhu D, Deng XW (2017). Noncanonical role of Arabidopsis COP1/SPA complex in repressing BIN2-mediated PIF3 phosphorylation and degradation in darkness. Proc Natl Acad Sci U S A.

[82] Alabadi D, Gallego-Bartolome J, Orlando L, Garcia-Carcel L, Rubio V, Martinez C, Frigerio M, Iglesias-Pedraz JM, Espinosa A, Deng XW, Blazquez MA (2008). Gibberellins modulate light signaling pathways to prevent Arabidopsis seedling de-etiolation in darkness. Plant J.

[83] de Lucas M, Daviere JM, Rodriguez-Falcon M, Pontin M, Iglesias-Pedraz JM, Lorrain S, Fankhauser C, Blazquez MA, Titarenko E, Prat S. A molecular framework for light and gibberellin control of cell elongation. Nature. 2008;451:480–4. 10.1038/nature06520 18216857

[84] Li K, Yu R, Fan LM, Wei N, Chen H, Deng XW (2016). DELLA-mediated PIF degradation contributes to coordination of light and gibberellin signalling in Arabidopsis. Nat Commun.

[85] Hennig L, Poppe C, Unger S, Schafer E. Control of hypocotyl elongation in Arabidopsis thaliana by photoreceptor interaction. Planta. 1999;208:257–63. 10.1007/s004250050557 10333589

[86] Leivar P, Monte E, Oka Y, Liu T, Carle C, Castillon A, Huq E, Quail PH (2008). Multiple phytochrome-interacting bHLH transcription factors repress premature seedling photomorphogenesis in darkness. Curr Biol.

[87] Rausenberger J, Hussong A, Kircher S, Kirchenbauer D, Timmer J, Nagy F, Schafer E, Fleck C. An integrative model for phytochrome B mediated photomorphogenesis: from protein dynamics to physiology. PLoS ONE. 2010;5:e10721. 10.1371/journal.pone.0010721 PMC2873432 20502669

[88] Soy J, Leivar P, Gonzalez-Schain N, Sentandreu M, Prat S, Quail PH, Monte E (2012). Phytochrome-imposed oscillations in PIF3 protein abundance regulate hypocotyl growth under diurnal light/dark conditions in Arabidopsis. Plant J.

[89] Soy J, Leivar P, Monte E (2014). PIF1 promotes phytochrome-regulated growth under photoperiodic conditions in Arabidopsis together with PIF3, PIF4, and PIF5. J Exp Bot.

[90] Van Buskirk EK, Reddy AK, Nagatani A, Chen M (2014). Photobody Localization of Phytochrome B Is Tightly Correlated with Prolonged and Light-Dependent Inhibition of Hypocotyl Elongation in the Dark. Plant Physiol.

[91] Jung JH, Domijan M, Klose C, Biswas S, Ezer D, Gao M, Khattak AK, Box MS, Charoensawan V, Cortijo S, Kumar M, Grant A, Locke JC, Schafer E, Jaeger KE, Wigge PA. Phytochromes function as thermosensors in Arabidopsis. Science. 2016;354:886–9. 10.1126/science.aaf6005 27789797

[92] Legris M, Klose C, Burgie ES, Rojas CC, Neme M, Hiltbrunner A, Wigge PA, Schafer E, Vierstra RD, Casal JJ. Phytochrome B integrates light and temperature signals in Arabidopsis. Science. 2016;354:897–900. 10.1126/science.aaf5656 27789798

[93] Bustin M (2001). Revised nomenclature for high mobility group (HMG) chromosomal proteins. Trends Biochem Sci.

[94] Catez F, Hock R (2010). Binding and interplay of HMG proteins on chromatin: Lessons from live cell imaging. Biochim Biophys Acta Gene Regul Mech.

[95] Ozturk N, Singh I, Mehta A, Braun T, Barreto G. HMGA proteins as modulators of chromatin structure during transcriptional activation. Front Cell Dev Biol. 2014;2:5. 10.3389/fcell.2014.00005. 10.3389/fcell.2014.00005 PMC4207033 25364713

[96] Aravind L, Landsman D (1998). AT-hook motifs identified in a wide variety of DNA-binding proteins. Nucleic Acids Res.

[97] Reeves R. Molecular biology of HMGA proteins: Hubs of nuclear function. Gene. 2001;277(1–2):63–81. 10.1016/s0378-1119(01)00689-8. 10.1016/s0378-1119(01)00689-8 11602345

[98] Cui TJ, Leng FF. Specific recognition of AT-Rich DNA sequences by the mammalian high mobility group-protein AT-hook 2: a SELEX study. Biochemistry. 2007;46:13059–66. 10.1021/Bi701269s. 10.1021/bi701269s 17956125

[99] Winter N, Nimzyk R, Bosche C, Meyer A, Bullerdiek J. Chromatin Immunoprecipitation to Analyze DNA Binding Sites of HMGA2. PLoS ONE. 2011;6:e18837. 10.1371/journal.pone.0018837. 10.1371/journal.pone.0018837 PMC3077414 21533145

[100] Bouallaga I, Massicard S, Yaniv M, Thierry F. An enhanceosome containing the Jun B/Fra-2 heterodimer and the HMG-I(Y) architectural protein controls HPV18 transcription. EMBO Rep. 2000;1:422–7. 10.1093/embo-reports/kvd091. 10.1093/embo-reports/kvd091 PMC1083764 11258482

[101] Bouallaga I, Teissier S, Yaniv M, Thierry F (2003). HMG-I(Y) and the CBP/p300 coactivator are essential for human papillomavirus type 18 enhanceosome transcriptional activity. Mol Cell Biol.

[102] Zhao K, Kas E, Gonzalez E, Laemmli UK. SAR-dependent mobilization of histone H1 by HMG-I/Y in vitro: HMG-I/Y Is Enriched in H1-depleted chromatin. EMBO J. 1993;12:3237–47. 10.1002/j.1460-2075.1993.tb05993.x PMC413591 8344261

[103] Catez F, Yang H, Tracey KJ, Reeves R, Misteli T, Bustin M. Network of dynamic interactions between histone H1 and high-mobility-group proteins in chromatin. Mol Cell Biol. 2004;24:4321–8. 10.1128/MCB.24.10.4321-4328.2004. 10.1128/MCB.24.10.4321-4328.2004 PMC400478 15121851

[104] Kishi Y, Fujii Y, Hirabayashi Y, Gotoh Y (2012). HMGA regulates the global chromatin state and neurogenic potential in neocortical precursor cells. Nat Neurosci.

[105] Li O, Vasudevan D, Davey CA, Droge P. High-level expression of DNA architectural factor HMGA2 and its association with nucleosomes in human embryonic stem cells. Genesis, 2006;44:523–9. 10.1002/dvg.20242. 10.1002/dvg.20242 17078040

[106] Narlikar GJ, Fan H, Kingston RE. Cooperation between complexes that regulate chromatin structure and transcription. Cell. 2002;108(4):475–87. 10.1016/s0092-8674(02)00654-2. 10.1016/s0092-8674(02)00654-2 11909519

[107] Ojolo SP, Cao S, Priyadarshani SVGN, Li W, Yan M, Aslam M, Zhao H, Qin Y. Regulation of plant growth and development: A review from a chromatin remodeling perspective. Front Plant Sci. 2018;9:1232. 10.3389/fpls.2018.01232. 10.3389/fpls.2018.01232 PMC6113404 30186301

[108] Knizewski L, Ginalski K, Jerzmanowski A (2008). Snf2 proteins in plants: Gene silencing and beyond. Trends Plant Sci.

[109] Cairns BR. The logic of chromatin architecture and remodelling at promoters. Nature. 2009;461:193–8. 10.1038/nature08450 19741699

[110] Clapier CR, Cairns BR (2009). The biology of chromatin remodeling complexes. Annu Rev Biochem.

[111] Hargreaves DC, Crabtree GR (2011). ATP-dependent chromatin remodeling: genetics, genomics and mechanisms. Cell Res.

[112] Narlikar GJ, Sundaramoorthy R, Owen-Hughes T. Mechanisms and functions of ATP-dependent chromatin-remodeling enzymes. Cell. 2013;154:490–503. 10.1016/j.cell.2013.07.011 PMC3781322 23911317

[113] Hu Y, Zhu N, Wang X, Yi Q, Zhu D, Lai Y, Zhao Y (2013). Analysis of rice Snf2 family proteins and their potential roles in epigenetic regulation. Plant Physiol Biochem.

[114] Han SK, Wu MF, Cui S, Wagner D. Roles and activities of chromatin remodeling ATPases in plants. Plant J. 2015;83(1):62–77. 10.1111/tpj.12877. 10.1111/tpj.12877 25977075

[115] Kumar SV, Wigge PA. H2A.Z-containing nucleosomes mediate the thermosensory response in Arabidopsis. Cell. 2010;140:136–47. 10.1016/j.cell.2009.11.006 20079334

[116] Coleman-Derr, D. Zilberman, D. Deposition of histone variant H2A.Z within gene bodies regulates responsive genes. PLoS Genet. 2012;8:e1002988. 10.1371/journal.pgen.1002988 PMC3469445 23071449

[117] Cortijo S, Charoensawan V, Brestovitsky A, Buning R, Ravarani C, Rhodes D, van Noort J, Jaeger KE, Wigge PA. Transcriptional Regulation of the Ambient Temperature Response by H2A.Z Nucleosomes and HSF1 Transcription Factors in Arabidopsis. Molecular Plant. 2017;10(10):1258–73. 10.1016/j.molp.2017.08.014. 10.1016/j.molp.2017.08.014 PMC6175055 28893714

[118] Tasset C, Yadav AS, Sureshkumar S, Singh R, van der Woude L, Nekrasov M, Tremethick D, van Zanten M, Balasubramanian S. POWERDRESS-mediated histone deacetylation is essential for thermomorphogenesis in Arabidopsis thaliana. PLOS Genetics. 2018;14(3). 10.1371/journal.pgen.1007280 10.1371/journal.pgen.1007280 PMC5874081 29547672

[119] van der Woude LC, Perrella G, Snoek BL, van Hoogdalem M, Novák O, van Verk MC, van Kooten HN, Zorn LE, Tonckens R, Dongus JA, Praat M, Stouten EA, Proveniers MCG, Vellutini E, Patitaki E, Shpulatov U, Kohlen W, Balasubramanian S, Ljung K, van der Krol AR, Smeekens S, Kaiserli E, van Zanten M. HISTONE DEACETYLASE 9 stimulates auxin-dependent thermomorphogenesis in Arabidopsis thaliana by mediating H2A.Z depletion. Proc Natl Acad Sci USA. 2019;116(50):25343–54. 10.1073/pnas.1911694116. 10.1073/pnas.1911694116 PMC6911240 31767749

[120] Cairns BR, Erdjument-Bromage H, Tempst P, Winston F, Kornberg RD (1998). Two Actin-Related Proteins Are Shared Functional Components of the Chromatin-Remodeling Complexes RSC and SWI/SNF. Mol Cell.

[121] Meagher RB, Deal RB, Kandasamy MK, Mckinney EC (2005). Nuclear Actin-Related Proteins as Epigenetic Regulators of Development. Plant Physiol.

[122] Fornara F, Panigrahi KC, Gissot L, Sauerbrunn N, Rühl M, Jarillo JA, Coupland G (2009). Arabidopsis DOF Transcription Factors Act Redundantly to Reduce CONSTANS Expression and Are Essential for a Photoperiodic Flowering Response. Dev Cell.

[123] Langmead B, Trapnell C, Pop M, Salzberg SL. Ultrafast and memory-efficient alignment of short DNA sequences to the human genome. Genome Biol. 2009;10(3). 10.1186/gb-2009-10-3-r25. 10.1186/gb-2009-10-3-r25 PMC2690996 19261174

[124] Robinson MD, McCarthy DJ, Smyth GK. edgeR: a Bioconductor package for differential expression analysis of digital gene expression data. Bioinformatics. 2010;26(1):139–40. 10.1093/bioinformatics/btp616 PMC2796818 19910308

[125] Zhang Y, Liu T, Meyer CA, Eeckhoute J, Johnson DS, Bernstein BE, Liu XS. Model-based Analysis of ChIP-Seq (MACS). Genome Biol. 2008;9(9). 10.1186/gb-2008-9-9-r137 10.1186/gb-2008-9-9-r137 PMC2592715 18798982

[126] Ramirez F, Ryan DP, Gruning B, Bhardwaj V, Kilpert F, Richter AS, Heyne S, Dundar F, Manke T (2016). deepTools2: a next generation web server for deep-sequencing data analysis. Nucleic Acids Res.

[127] Robinson JT, Thorvaldsdóttir H, Winckler W, Guttman M, Lander ES, Getz G, Mesirov JP (2011). Integrative genomics viewer. Nat Biotechnol.

[128] Thorvaldsdottir H, Robinson JT, Mesirov JP (2013). Integrative Genomics Viewer (IGV): High-performance genomics data visualization and exploration. Brief Bioinform.

[129] Machanick P, Bailey TL. MEME-ChIP: motif analysis of large DNA datasets. Bioinformatics. 2011;27:1696–7. 10.1093/bioinformatics/btr189 PMC3106185 21486936

[130] Heinz S, Benner C, Spann N, Bertolino E, Lin YC, Laslo P, Cheng JX, Murre C, Singh H, Glass CK. Simple Combinations of Lineage-Determining Transcription Factors Prime cis-Regulatory Elements Required for Macrophage and B Cell Identities. Mol Cell. 2010;38(4):576–89. 10.1016/j.molcel.2010.05.004. 10.1016/j.molcel.2010.05.004 PMC2898526 20513432

[131] Maere S, Heymans K, Kuiper M. BiNGO: A Cytoscape plugin to assess overrepresentation of Gene Ontology categories in Biological Networks. Bioinformatics, 2005;21(16):3448–9. 10.1093/bioinformatics/bti551. 10.1093/bioinformatics/bti551 15972284

[132] Howe EA, Sinha R, Schlauch D, Quackenbush J. RNA-Seq analysis in MeV. Bioinformatics. 2011;27(22):3209–10. 10.1093/bioinformatics/btr490. 10.1093/bioinformatics/btr490 PMC3208390 21976420

