# Supplementary material for: SUPPRESSOR OF PHYTOCHROME B-4 #3 reduces the expression of PIF-activated genes and increases expression of growth repressors to regulate hypocotyl elongation in short days
Source: BMC Plant Biol. 2022 Aug 15;22:399. doi: 10.1186/s12870-022-03737-z (PMC9377115; doi:10.1186/s12870-022-03737-z)
Supplement: Supplementary file 2 — Additional file 2: Supplementary Table 2. The fold change of PIF-target genes from Figure 2. Logfc indicates log2 difference in gene expression between sob3-6 and SOB3-D seedlings. Highlighted values denote transcripts that are differentially regulated between the two genotypes for the given time point as indicated by a p-value (FDR-adjusted p-value) < 0.05 [file 12870_2022_3737_MOESM2_ESM.pdf]

		ZT4		ZT9		ZT24	
		logfc	p-value	logfc	p-value	logfc	p-value
BR6OX2	AT3G30180.1	0.52529746	0.00776614	0.13840925	0.77265736	0.83221641	1.08E-06
CDF5	AT1G69570.1	0.31443069	0.59513017	1.22135107	2.00E-05	0.87045383	0.00058549
CPD	AT5G05690.1	0.51621402	0.00129225	0.0700124	0.91052375	0.19553694	0.51054218
	AT5G05690.2	0.49821662	0.00170267	0.07483457	0.88963439	0.20943344	0.43548527
	AT5G05690.3	0.51004007	0.00157636	0.07855773	0.88071435	0.17367329	0.59611293
HFR1	AT1G02340.1	-0.4738601	0.09525665	0.75349261	0.00560628	0.06308434	1
IAA19	AT3G15540.1	1.06124571	0.00086698	1.72757759	1.19E-08	2.09401133	1.22E-16
IAA29	AT4G32280.1	2.87870697	1.72E-16	1.48360599	5.77E-06	1.8413584	1.09E-12
PIL1	AT2G46970.1	1.33030748	0.00172431	1.99978423	7.82E-06	1.95293317	4.44E-29
PRE1	AT5G39860.1	0.93639883	0.01146536	1.24465456	0.02568388	0.82985223	0.03177243
SAUR19	AT5G18010.1	1.02237269	0.00097825	1.42263883	0.00645015	0.69840737	0.01039373
SAUR22	AT5G18050.1	2.89532143	1.31E-13	2.38587574	0.0002006	1.51594787	2.90E-09
SAUR24	AT5G18080.1	1.14751726	0.0127236	0.75359945	0.61289263	1.02194354	0.01669815
XTR7	AT4G14130.1	0.83395381	0.2098648	2.41535766	2.35E-07	1.24930662	0.02581414
YUC8	AT4G28720.1	1.76739828	1.05E-08	1.83377427	2.56E-07	2.29549735	6.77E-17

Supplementary Table 2: The fold change of *PIF*-target genes from Figure 2. Logfc indicates $\log_2$ difference in gene expression between *sob3-6* and *SOB3-D* seedlings. Highlighted values denote transcripts that are differentially regulated between the two genotypes for the given time point as indicated by a p-value (FDR-adjusted p-value) < 0.05.
