# Supplementary material for: SUPPRESSOR OF PHYTOCHROME B-4 #3 reduces the expression of PIF-activated genes and increases expression of growth repressors to regulate hypocotyl elongation in short days
Source: BMC Plant Biol. 2022 Aug 15;22:399. doi: 10.1186/s12870-022-03737-z (PMC9377115; doi:10.1186/s12870-022-03737-z)
Supplement: Supplementary file 4 — Additional file 4: Supplementary Table 4. Binding of SOB3, based on the ChIP-seq data, to AHL-repressed or AHL-induced genes named in Fig. 2B or 2C, respectively. * = bound by SOB3 in one rep; ** = bound by SOB3 in two reps; *** = bound by SOB3 in three reps. Blue highlighting indicates the gene is counted as a SOB3-bound gene at that time point (i.e. it was bound in at least 2 ChIP-seq reps). “binds uORF” indicates that although annotation using HOMER did not detect the gene as bound by SOB3 (i.e. the gene’s TSS was not the closest TSS to any SOB3 ChIP-seq peak), a uORF located just upstream of the gene was identified as SOB3-bound. [file 12870_2022_3737_MOESM4_ESM.pdf]

**Logfc+ ZT4 (repressed)**	**+ ZT9**	**+ZT24**	**Logfc- ZT4 (induced)**	**-ZT9**	**-ZT24**
SAUR19** (AT5G18010)	SAUR19*	SAUR19***	ELF3*** (AT2G25930)	ELF3***	ELF3***
SAUR22** (AT5G18050)	SAUR22*	SAUR22***	ELF4 (binds uORF in promoter of ELF4***) (AT2G40080)	ELF4***	ELF4 (binds uORF in promoter of ELF4***)
SAUR24*** (AT5G18080)	SAUR24**	SAUR24***	HY5*** (AT5G11260)	HY5**	HY5***
PRE1*** (AT5G39860)	PRE1***	PRE1***	BBX21*** (AT1G75540)	BBX21***	BBX21***
CPD*** (AT5G05690)	CPD***	CPD***	BBX22*** (AT1G78600)	BBX22***	BBX22***
BES1*** (AT1G19350)	BES1***	BES1***	IAA17*** (AT1G04250)	IAA17***	IAA17***
YUC8*** (AT4G28720)	YUC8***	YUC8***	PRR9 (binds promoter in uORF of PRR9***) (AT2G46790)	PRR9 (binds promoter in uORF of PRR9***)	PRR9 (binds promoter in uORF of PRR9***)
PIF4*** (AT2G43010)	PIF4***	PIF4***	RGA*** (AT2G01570)	RGA***	RGA***
PIF5*** (AT3G59060)	PIF5***	PIF5***			
PIF7*** (AT5G61270)	PIF7***	PIF7***			
PIL1*** (AT2G46970)	PIL1***	PIL1***			
BR6OX2*** (AT3G30180)	BR6OX2***	BR6OX2***			
IAA19*** (AT3G15540)	IAA19***	IAA19***			
IAA29*** (AT4G32280)	IAA29***	IAA29***			
CDF5*** (AT1G69570)	CDF5***	CDF5***			
PIF8*** (AT4G00050)	PIF8***	PIF8***			
HFR1*** (AT1G02340)	HFR1***	HFR1***			
XTR7*** (AT4G14130)	XTR7***	XTR7***			

*=bound by SOB3 in one rep

**=bound by SOB3 in two reps

***=bound by SOB3 in three reps

Counted as a SOB3-bound gene for the indicated time point (bound in at least 2 reps)

Supplementary Table 4: Binding of SOB3, based on the ChIP-seq data, to AHL-repressed or AHL-induced genes named in Fig. 2B or 2C, respectively.

* = bound by SOB3 in one rep; ** = bound by SOB3 in two reps; *** = bound by SOB3 in three reps. Blue highlighting indicates the gene is counted as a SOB3-bound gene at that time point (i.e. it was bound in at least 2 ChIP-seq reps). “binds uORF” indicates that although annotation using HOMER did not detect the gene as bound by SOB3 (i.e. the gene’s TSS was not the closest TSS to any SOB3 ChIP-seq peak), a uORF located just upstream of the gene was identified as SOB3-bound.
