# Supplementary material for: SUPPRESSOR OF PHYTOCHROME B-4 #3 reduces the expression of PIF-activated genes and increases expression of growth repressors to regulate hypocotyl elongation in short days
Source: BMC Plant Biol. 2022 Aug 15;22:399. doi: 10.1186/s12870-022-03737-z (PMC9377115; doi:10.1186/s12870-022-03737-z)
Supplement: Supplementary file 5 — Additional file 5: Supplementary Figure 1. Binding of SOB3 to loci associated with BES1 (A), CPD (B), BBX22 (C), or PIF4 (D) based on the ChIP-seq data generated from ProSOB3::SOB3-GFP sob3-4 seedlings harvested at ZT4, ZT9, or ZT24. Plots show relative fold enrichment in the ChIP samples compared to their respective input controls. [file 12870_2022_3737_MOESM5_ESM.pdf]

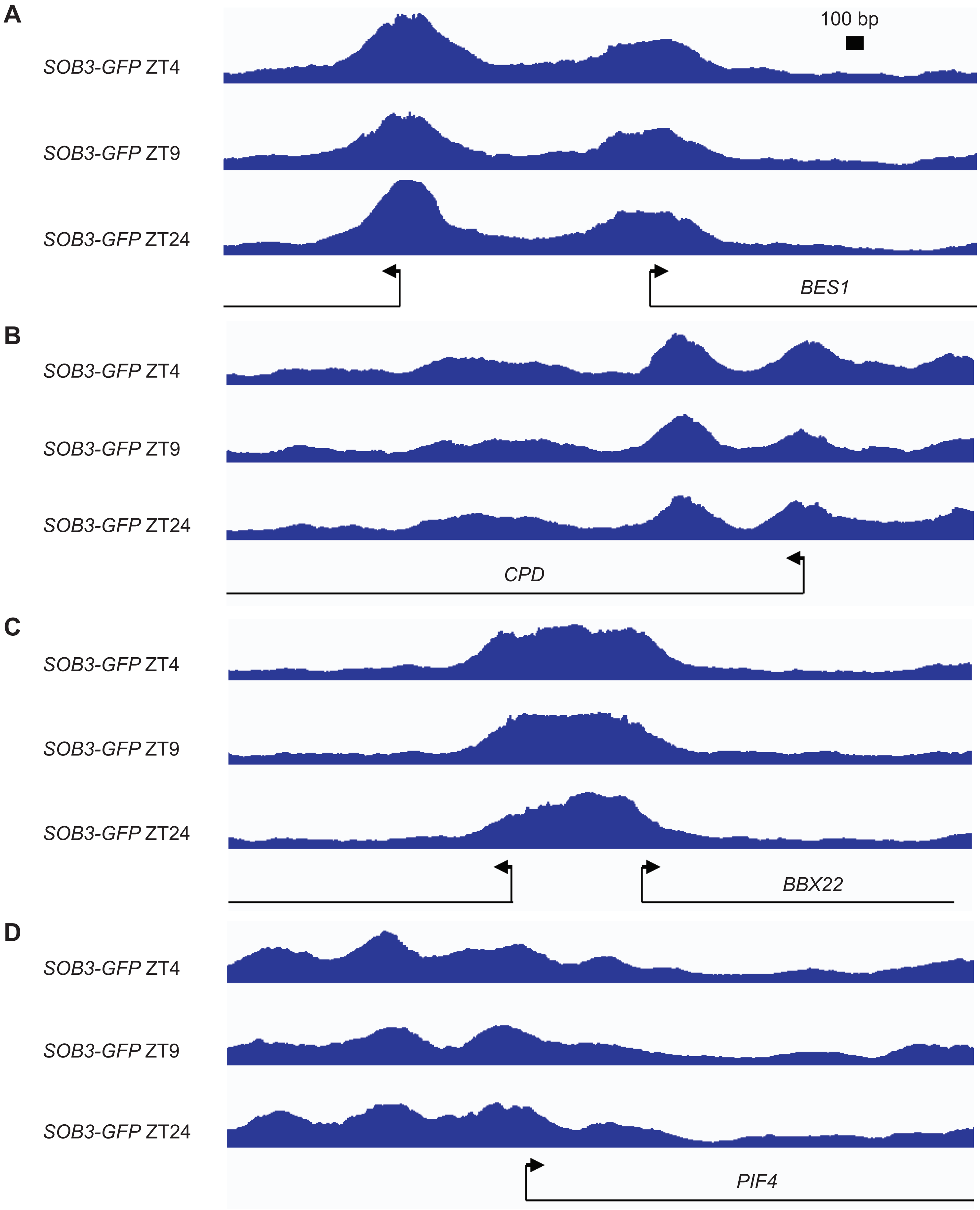

Supplementary Figure 1: Binding of SOB3 to loci associated with *BES1* **(A)**, *CPD* **(B)**, *BBX22* **(C)**, or *PIF4* **(D)** based on the ChIP-seq data generated from *ProSOB3::SOB3-GFP sob3-4* seedlings harvested at ZT4, ZT9, or ZT24. Plots show relative fold enrichment in the ChIP samples compared to their respective input controls.
