# Supplementary material for: SUPPRESSOR OF PHYTOCHROME B-4 #3 reduces the expression of PIF-activated genes and increases expression of growth repressors to regulate hypocotyl elongation in short days
Source: BMC Plant Biol. 2022 Aug 15;22:399. doi: 10.1186/s12870-022-03737-z (PMC9377115; doi:10.1186/s12870-022-03737-z)
Supplement: Supplementary file 6 — Additional file 6: Supplementary Figure 2. Relative binding of SOB3, based on the ChIP-seq data generated from ProSOB3::SOB3-GFP sob3-4 seedlings harvested at ZT4, ZT9, or ZT24, to genes identified as induced or repressed by AHLs at only a single time point from the RNA-seq data for SOB3-D and sob3-6. (A) Relative binding of SOB3 to genes identified as repressed (left) or induced (right) by AHLs only at ZT4. (B) Relative binding of SOB3 to genes identified as repressed (left) or induced (right) by AHLs only at ZT9. (C) Relative binding of SOB3 to genes identified as repressed (left) or induced (right) by AHLs only at ZT24. [file 12870_2022_3737_MOESM6_ESM.pdf]

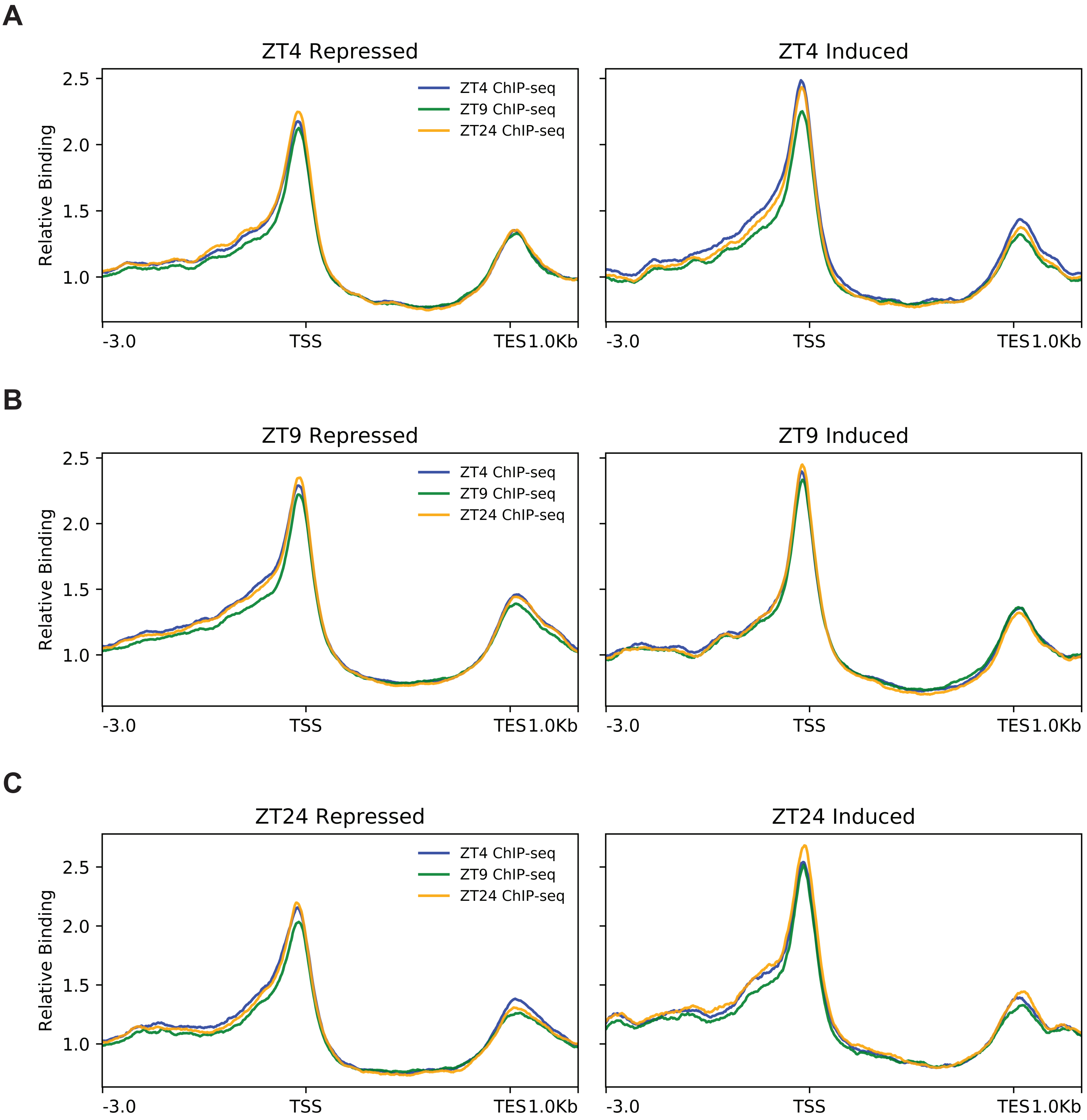

Supplementary Figure 2: Relative binding of SOB3, based on the ChIP-seq data generated from *ProSOB3::SOB3-GFP sob3-4* seedlings harvested at ZT4, ZT9, or ZT24, to genes identified as induced or repressed by AHLs at only a single time point from the RNA-seq data for *SOB3-D* and *sob3-6*.

(A) Relative binding of SOB3 to genes identified as repressed (left) or induced (right) by AHLs only at ZT4.

(B) Relative binding of SOB3 to genes identified as repressed (left) or induced (right) by AHLs only at ZT9.

(C) Relative binding of SOB3 to genes identified as repressed (left) or induced (right) by AHLs only at ZT24.
