# Supplementary material for: SUPPRESSOR OF PHYTOCHROME B-4 #3 reduces the expression of PIF-activated genes and increases expression of growth repressors to regulate hypocotyl elongation in short days
Source: BMC Plant Biol. 2022 Aug 15;22:399. doi: 10.1186/s12870-022-03737-z (PMC9377115; doi:10.1186/s12870-022-03737-z)
Supplement: Supplementary file 7 — Additional file 7: Supplementary Figure 3. Relative binding of SOB3, based on the ChIP-seq data generated from ProSOB3::SOB3-GFP sob3-4 seedlings harvested at ZT4, ZT9, or ZT24, to genes identified as induced or repressed by AHLs at only two time points from the RNA-seq data for SOB3-D and sob3-6. (A) Relative binding of SOB3 to genes identified as repressed (left) or induced (right) by AHLs at ZT4 and ZT9 but not at ZT24. (B) Relative binding of SOB3 to genes identified as repressed (left) or induced (right) by AHLs at ZT4 and ZT24 but not at ZT9. (C) Relative binding of SOB3 to genes identified as repressed (left) or induced (right) by AHLs at ZT9 and ZT24 but not at ZT4. [file 12870_2022_3737_MOESM7_ESM.pdf]

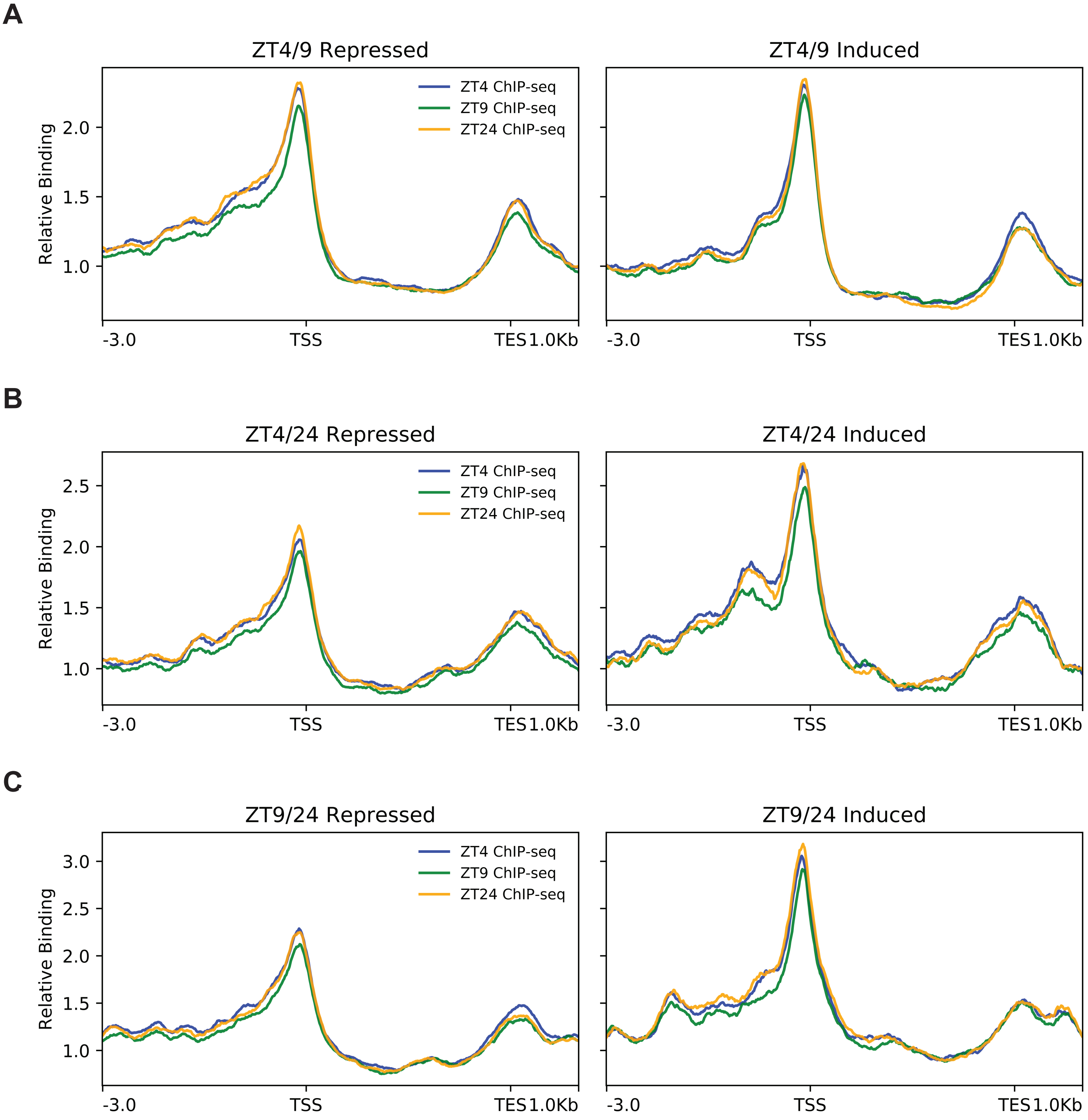

Supplementary Figure 3: Relative binding of SOB3, based on the ChIP-seq data generated from *ProSOB3::SOB3-GFP sob3-4* seedlings harvested at ZT4, ZT9, or ZT24, to genes identified as induced or repressed by AHLs at only two time points from the RNA-seq data for *SOB3-D* and *sob3-6*.

(A) Relative binding of SOB3 to genes identified as repressed (left) or induced (right) by AHLs at ZT4 and ZT9 but not at ZT24.

(B) Relative binding of SOB3 to genes identified as repressed (left) or induced (right) by AHLs at ZT4 and ZT24 but not at ZT9.

(C) Relative binding of SOB3 to genes identified as repressed (left) or induced (right) by AHLs at ZT9 and ZT24 but not at ZT4.
